# Quasi-continuous cotranslational compaction and folding of a multidomain protein

**DOI:** 10.1038/s41467-026-78090-y

**Published:** 2026-10-05

**Authors:** Spyridoula Mitsikosta, Justin M. Westerfield, Fátima Pardo-Avila, Michael Levitt, Gunnar von Heijne, Ane Metola

**Affiliations:** 1 Department of Biochemistry and Biophysics, Stockholm University, Stockholm, Sweden; 2 Department of Structural Biology, Stanford University, Palo Alto, CA USA; 3 Science for Life Laboratory Stockholm University, Box 1031, Solna, Sweden

**Keywords:** Protein folding, Computational biophysics, Cryoelectron microscopy, Single-molecule biophysics

## Abstract

Most proteins start to fold cotranslationally as they come off the ribosome. So far, studies of cotranslational folding have focused mainly on small, single-domain proteins. Here, we have used Force Profile Analysis to study the cotranslational folding of Firefly Luciferase, a complex 550-residue protein composed of an N-terminal domain (NTD) encompassing two split Rossmann folds (RF-1, RF-2) and a β-roll, and a flexibly attached C-terminal domain (CTD). The folding process is characterized by a quasi-continuous series of compaction/folding steps that generate intermediate-size pulling forces on the nascent chain, punctuated by a prominent high-force event that represents the folding of the RF-2 domain, and a few low-force instances that likely indicate the formation of distinct folding intermediates. Trigger Factor interacts extensively with the nascent chain when the central part of RF-2 and the early parts of the CTD are synthesized. Our analysis uncovers a cotranslational compaction/folding process that is rich in detail and not just a simple succession of a few distinct, cooperative folding transitions.

## Abstract (web summary)

Force Profile Analysis show that firefly luciferase folds co-translationally through a continuous compaction process, punctuated by a major subdomain folding event. Trigger Factor binds to the nascent chain at distinct steps in the folding profile.

## Introduction

While it has long been acknowledged that many proteins may begin to fold cotranslationally in vivo, it is only relatively recently that a range of methodological advances have opened up this area to more detailed studies. Early work mainly used assays based on protease resistance^1,2^, while today, powerful new biochemical and biophysical methods such as force-profile analysis (FPA)^3,4^, pulse-proteolysis^5^, NMR^6^, hydrogen-deuterium exchange mass-spectrometry (HDX-MS)^7^, cryo-electron microscopy (cryo-EM)^3^, optical tweezers^8^, fluorescence-resonance energy transfer^9^, and computational approaches^10^ have advanced the field to a new level.

Thus, over the past ~10 years, it has been shown that small protein domains (up to ~100 residues in size) can fold wholly or in part inside the ribosome exit tunnel (ET)^11,12^, that unfolded protein domains held in proximity to the ribosome can interact non-specifically with the surface of the ribosome and that on-ribosome folding reduces the entropic penalty of folding and promotes formation of partially folded intermediates^13,14^, that folding pathways can differ dramatically when folding happens on-ribosome as compared to off-ribosome^15^, and that a large proportion of proteins likely start to fold cotranslationally^16^. Moreover, cotranslational nascent chain—chaperone interactions have been shown to play important roles during protein folding in vivo^17^.

**Fig. 1:**
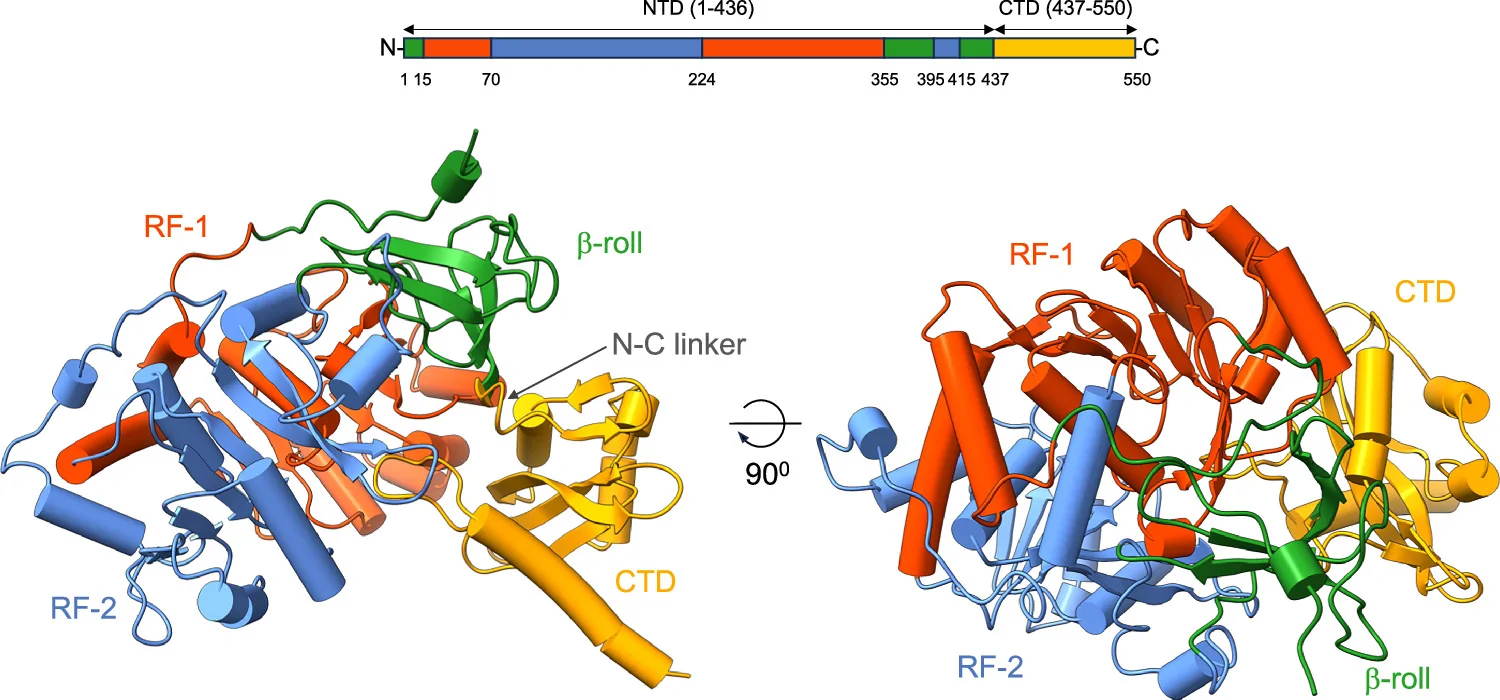
FLuc domain structure. FLuc 3D structure (AlphaFold DB^71^ AF-P08659-F1-v4, see Supplementary Fig. 9 for pLDDT and PAE values). The two Rossmann folds are colored in orange (RF-1) and blue (RF-2), the β-roll domain in green, and the C-terminal domain (CTD) in yellow. Approximate (sub)domain boundaries are indicated at the top.

Many of these studies have focused on single-domain proteins that are small enough to be able to fold inside the ET, or in the vestibule that defines the exit port at the distal end of the ET. Larger proteins, however, especially those that are composed of identifiable but tightly interacting or interdigitating subdomains, are more challenging and only a handful of such studies have been published to date^2,4,7,18–23^. We have now applied FPA to one such protein, Firefly Luciferase (FLuc), a 550-residue protein that has been much studied in the context of chaperone-mediated folding^24–27^. FLuc is composed of a large N-terminal domain (NTD) encompassing two split Rossmann folds (RF-1, RF-2) and a β-roll, and a flexibly attached C-terminal α/β domain (CTD), Fig. 1. FLuc folds rapidly and efficiently during in vitro translation in an *E. coli* lysate even in the absence of chaperones^28^, but requires chaperones to refold from a denatured state^29^. Cotranslational folding of FLuc was previously proposed to proceed via the formation of an N-terminal protease-resistant 21 kDa intermediate^2^, and a recent HDX-MS study has shown that, when synthesized on *E. coli* ribosomes, FLuc adopts a close-to-native structure before, or at the latest at, the time when the NTD has emerged from the ET^22^.

Here, we report a high-resolution FPA study of the cotranslational folding of FLuc. We find that FLuc undergoes what we suggest is a quasi-continuous series of cotranslational compaction/folding steps, punctuated by a prominent folding event and a few quiescent periods that likely correspond to the formation of distinct folding intermediates. The chaperone Trigger Factor (TF) strongly affects the folding of a portion of the first half of the NTD and of an early portion of the CTD, but otherwise has no or only highly localized effects. The quasi-continuous character of the folding process raises questions about what should be considered a cotranslational folding intermediate and how such intermediates may relate to cooperative folding events during translation.

## Results

### Force profile analysis

FPA is based on the original discovery^30^ of translational arrest peptides (APs) – short polypeptide sequences in nascent chains (NCs) that make specific interactions with ribosomal rRNA in the vicinity of the peptidyl transferase center (PTC) and thereby induce slight changes in the relative orientation of the A- and P-site tRNAs that lead to strong reductions in the rate of transpeptidation^31^. An external pulling force acting on the AP can dislodge it from its binding site in the ET and thereby restore the normal translation rate^32–34^; APs can thus be used as force sensors to report on various cotranslational processes that generate force on the AP, such as protein folding^3^, membrane protein biogenesis^35^, or protein translocation^36^. Exceptionally strong APs can even be used to irreversibly stall a NC on the ribosome^37^, making it possible to purify stable ribosome-nascent chain complexes (RNCs) for analysis by techniques such as cryo-EM^3^, NMR^13^, and HDX-MS^22^.

**Fig. 2:**
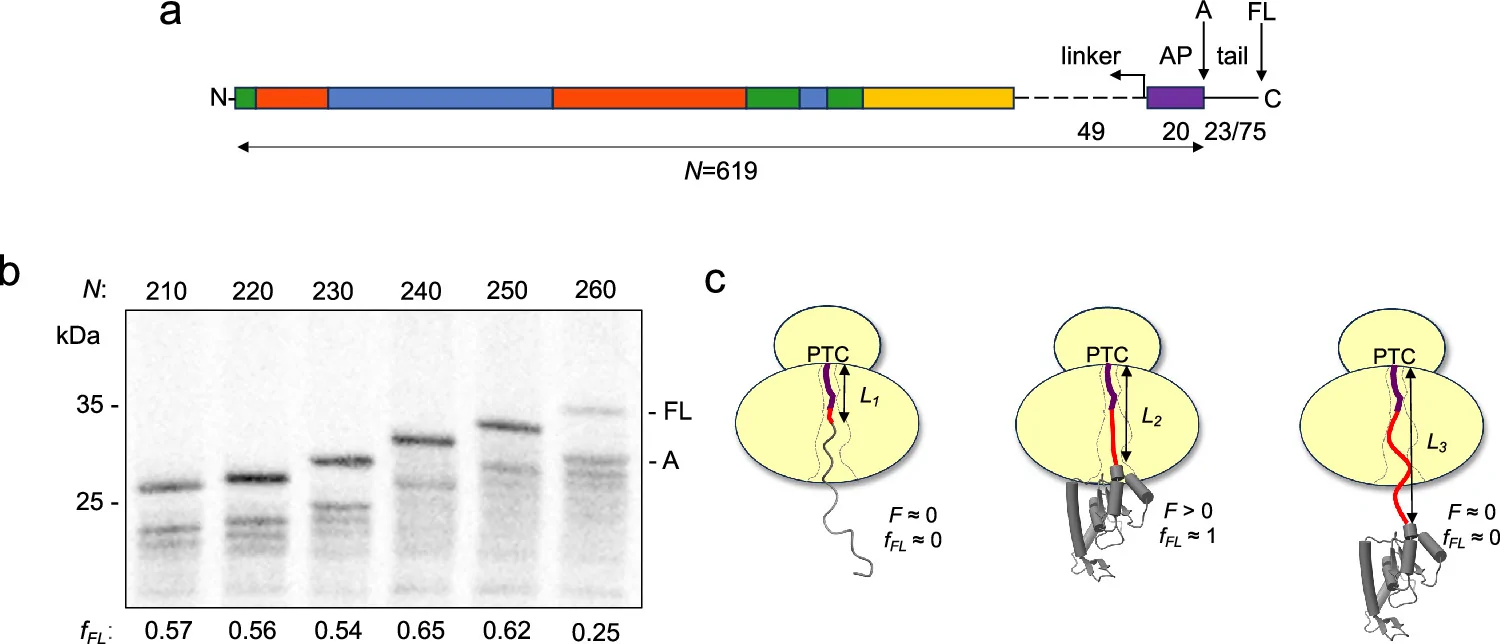
Force Profile Analysis of FLuc. **a** Construct design. In the construct shown (*N* = 619 residues), full-length FLuc (coloring as in Fig. 1) is fused to a 49-residue GS-linker, the 20-residue SecM(*Ec*) AP (SGSFSTPVWISQAQGIRAGP), and a 75-residue C-terminal tail; in constructs with *N* ≤ 250 residues, the C-terminal tail is 23 residues long (see Supplementary Data 1 for sequences). The full set of constructs was made by deleting 5 residues at a time starting from the C-terminal end of the 49-residue GS-linker (left-pointing arrow); the shortest construct (*N* = 70 residues) contains FLuc residues 1–50, the 20-residue SecM(*Ec*) AP, and a 23-residue tail. The 20-residue AP is constant in all constructs. Arrested (*A*) and full-length (*FL*) products are indicated. **b** Example SDS-PAGE gel showing constructs *N* = 210-260 residues. The fraction full-length protein is calculated as *f*_*FL*_ = *I*_*FL*_/(*I*_*FL*_ + *I*_*A*_), where *I*_*FL*_ and *I*_*A*_ are the intensities of the *FL* and *A* bands, respectively. The bands immediately below the *A* product are always seen in these types of experiments and most likely indicate one or more ribosomes stacked behind the arrested ribosome. Faint bands seen at lower Mw do not vary in size between the different constructs and hence represent unrelated background bands or weak translational pause sites^87^. **c** Cartoon model illustrating how cotranslational folding of a protein domain generates force on the AP. In constructs with a short linker (red), the C-terminal end of the domain is located deep inside the ET at the point when the ribosome reaches the C-terminal end of the AP (purple), there is not enough room for the domain to fold, and hence little force is generated (*F* ≈ 0, *f*_*FL*_ ≈ 0; left). At intermediate linker lengths, the domain starts to fold at the point when the ribosome reaches the C-terminal end of the AP, generating force on the AP by stretching the NC (*F* > 0, *f*_*FL*_ ≈ 1; middle). Finally, in constructs with long linkers, the domain has already folded at the point when the ribosome reaches the C-terminal end of the AP, and again no force is generated (*F* ≈ 0, *f*_*FL*_ ≈ 0; right). The full gel for panel *b* and *f*_*FL*_ values *f*or *n* = 3 repeats of these constructs is provided in the Source Data file.

In a typical FPA experiment^3^, an intermediate-strength AP (e.g., the *E. coli* SecM(*Ec*) AP) is fused via a linker to the C-terminal end of the target protein, and a C-terminal tail is added behind the AP, Fig. 2a. A series of constructs is made by truncating one or a few residues at a time from the C-terminal end of the linker sequence and continuing into the target protein. Each construct is then translated and pulse-labeled with [^35^S]-Met for a set time, either in vivo or in an in vitro translation system such as PURExpress™, and analyzed by SDS-PAGE, Fig. 2b. The bands corresponding to arrested (*A*) and full-length (*FL*) protein are quantitated on a Phosphorimager, and the fraction full-length protein (*f*_*FL*_ = *I*_*FL*_/(*I*_*FL*_ + *I*_*A*_), where *I*_*A*_ and *I*_*FL*_ are the measured intensities of the respective bands) is calculated for each construct. *f*_*FL*_ serves as a convenient proxy for the pulling force acting on the AP, and can in principle be converted into force measured in pN^38^; the higher the pulling force, the higher is *f*_*FL*_.

Finally, a force profile (FP) is obtained by plotting  *f*_*FL*_ against *N*, the number of residues between the N-terminal end of the construct and the C-terminal end of the AP. In the case of cotranslational protein folding, peaks in the FP correspond to constructs in which a (sub)domain can just reach a region in or immediately outside the ET where there is enough space for it to fold^3,11^, Fig. 2c. Some of the free energy of folding will thus be stored as elastic energy in the linker (red in Fig. 2c) that attaches the subdomain to the AP, generating a pulling force on the AP and a high *f*_*FL*_ value^39^.

FPA does not follow folding in real time, but infers folding events from variations in the pulling force exerted on the AP in progressively longer constructs. With a typical in vivo translation rate of ~10 codons per second (and ~1 codon per second in the PURE in vitro system), the NC has ≳100 msec to equilibrate between folded and unfolded conformers during each translation step, which is long compared to typical folding rates for single-domain proteins (μsec-msec). Kinetic in vitro experiments have shown that FLuc is already folded into an active conformation when released from the *E. coli* ribosome^28^, supporting a rapid folding rate. The FLuc FP should thus provide a good approximation to the progressive real-time evolution of folding/unfolding equilibration during translation. Of note, because the SecM(*Ec*) AP does not noticeably stall translation during high pulling-force conditions^3^, high peaks in the FP are generated by constructs that are translated at normal translation rates from beginning to end.

### A FLuc FP at five-residue resolution

A set of FLuc constructs was prepared starting from a design with full-length FLuc (residues 1–550) fused to a 49-residue Gly-Ser (GS) linker, a 20-residue SecM(*Ec*) AP (SGSFSTPVWISQAQGIRAGP; note the SGS tripeptide included with the AP to “insulate” the AP from FLuc), and a 75-residue C-terminal tail, Fig. 2a. Shorter constructs were designed by deleting five residues at a time starting from the C-terminal end of the 49-residue GS-linker and continuing until only FLuc residues 1–50 remained; constructs with *N* ≤ 250 residues were designed with a shorter, 23-residue C-terminal tail. All in all, the full set consists of 128 constructs with each construct referred to by its *N*-value, *i.e*., the number of residues from the N-terminal end of FLuc to the C-terminal Pro residue in the AP. All constructs were transcribed and translated in vitro in the *E. coli*-derived PURExpress™ system, and analyzed by SDS-PAGE.

**Fig. 3:**
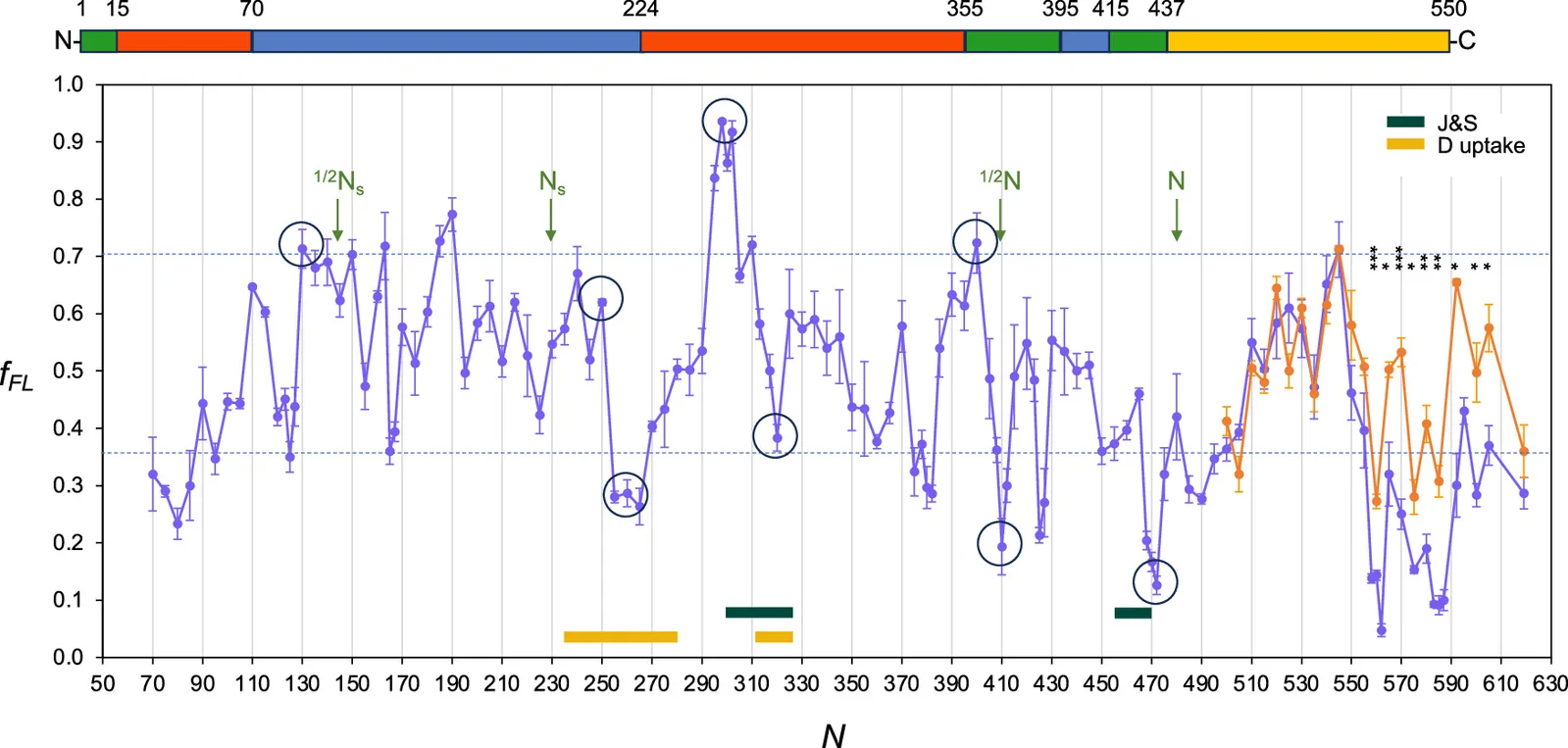
FLuc force profile. Average *f*_*FL*_ values plotted against *N* for FLuc constructs (in blue), and for the CTD expressed by itself (orange).Peaks and dips discussed in the main text are encircled. Subdomains are shown above the FP (colored as in Fig. 1), cotranslational folding intermediates predicted by the Jacobs and Shakhnovich (J&S) method^44^ are indicated by dark green bars, and peptides showing increased deuterium uptake (D uptake) compared to folded full-length FLuc in the stalled ^1/2^N construct^22^ are indicated by yellow bars; in all cases, *N-*values for the subdomain boundaries shown above the FP and the predicted folding intermediates were calculated by adding 40 residues to the corresponding FLuc residue numbers (*e.g*., the predicted folding intermediate ending at FLuc residues 260–285, Supplementary Fig. 1, is plotted at *N* = 300–325 residues). Four constructs previously analyzed by HDX-MS^22^ are indicated by their names (^1/2^N_s_, N_s_, ^1/2^N, N; in green). All data points represent averages for *n* ≥ 3 biological replicates, with the exact *n* values given in the Source Data file. Significant differences between the FLuc and CTD data are indicated by *p*-values calculated by a two-sided Student’s *t* test (*p* < 0.05: *; *p* < .01: **, *p* < .001: ***). Error bars indicate the standard error of the mean (SEM). Source data are provided in the Source Data file.

The resulting FLuc FP, obtained at five-residue resolution (and two-residue resolution around a few peaks and dips), is shown in Fig. 3 (blue curve). The FP extends from *N* = 70 residues (FLuc residues 1–50 fused to the 20-residue AP and a 23-residue C-terminal tail) to *N* = 619 (FLuc residues 1–550 fused via a 49-residue GS-linker to the 20-residue AP and a 75-residue C-terminal tail). While the 20-residue AP is common to all constructs and occupies the proximal part of the ribosome exit tunnel (ET) at the point of translational arrest, the ~20 residues located in the vestibule of the ET differ in sequence between different constructs and could in principle affect *f*_*FL*_. However, no residues in this region of the SecM protein have so far been implicated in contributing to translational arrest^40^, although hydrogen bond interactions between large polar residues and tunnel components can be seen in cryo-EM structures^41^, possibly giving rise to minor variations in *f*_*FL*_.

The FP is characterized by stretches with many small peaks and dips of intermediate amplitude (0.35 ≲ *f*_*FL*_ ≲ 0.7) punctuated by a few high peaks and deep dips. Previous work on a range of proteins has shown that cooperative folding events happening in or just outside the ET give rise to distinct, high-amplitude peaks in the FP^3,4,11,20,38,39,42,43^. Conversely, we hypothesize that deep dips in the FP correspond to points during translation where a (semi)stable folding intermediate has just been formed while the following part of the nascent chain (NC) has not yet grown sufficiently in size to re-initiate folding. The intermediate-amplitude, rapidly varying pattern seen over large regions of the FLuc FP has not been seen before in single-domain proteins FPs, and we propose that it represents quasi-continuous compaction (rather than cooperative folding) of the NC as it emerges from the ET.

Because the ET sequesters ~40 residues of the NC^41^, and protein domains composed of more than ~120 amino acids typically start to fold when their C-terminus is ~40 residues away from the peptidyl-transferase center (PTC) in the ribosome^1,11^, a given FLuc (sub)domain ending at FLuc residue *n* is expected to fully emerge from the ET at an *N*-value *N* ≈ *n* + 40 residues. To facilitate the interpretation of the FP, we thus aligned the FLuc domain structure schematic shown in Fig. 1 to the FP by adding 40 residues to the (sub)domain boundaries (*e.g*., the NTD, which ends at FLuc residue 437, is aligned at *N* = 477 residues), such that the boundaries match the *N*-values at which they are expected to emerge from the ET. We further used the Jacobs & Shakhnovich method^44^ to predict the formation of potential cotranslational folding intermediates, Supplementary Fig. 1. As indicated in Fig. 3, the two predicted folding intermediates (ending at FLuc residues 260–285 and 415–430) are expected to emerge from the ET at *N* = 300–325 and 455–470 residues (dark green bars).

The first main peak at *N* = 130–150 residues, and the first major dip at *N* = 255–265 residues, suggest that some degree of compaction/folding commences approximately when FLuc residues 1–90 have emerged outside the ET and continues until what appears to be a compact subdomain comprising residues 1–215 ( ± 5 residues) has formed outside the ET, Fig. 3. Available HDX-MS data for stalled *E. coli* FLuc RNCs corresponding to constructs *N* = 145 (^1/2^N_S_, Fig. 3) and *N* = 230 (N_S,_ Fig. 3) indicate that the parts exposed outside the ribosome have not adopted the native structure in the former, and that native-like structure is seen only for FLuc residues ~160–190 in the latter^22^. While early work demonstrated the cotranslational formation of a protease-resistant 21 kDa FLuc intermediate comprising residues 1–190^2^; a recent study has shown that this may represent a non-native homodimer produced during the proteolysis experiment^22^. We thus propose that FLuc residues 1–215 ( ± 5) define an early compacted folding intermediate with some native-like structure.

The prominent dip in the FP at *N* = 255–265 residues indicates that the segment containing FLuc residues ~215–225 does not fold onto the 1–215 folding intermediate, but rather remains in a flexible conformation as the NC elongates. Interestingly, HDX-MS data^22^ for the stalled ^1/2^N construct (corresponding to our *N* = 410 construct) shows increased deuterium uptake compared to folded full-length FLuc for peptides covering FLuc residues 195–238 and 272–287 (yellow bars, Fig. 3), matching the dips in the FP at *N* = 255–265 and 315–325 residues. Thus, FLuc residues ~215–225 and ~275–285 remain in a flexible conformation at least until translation has proceeded to a NC-length of 410 residues.

**Fig. 4:**
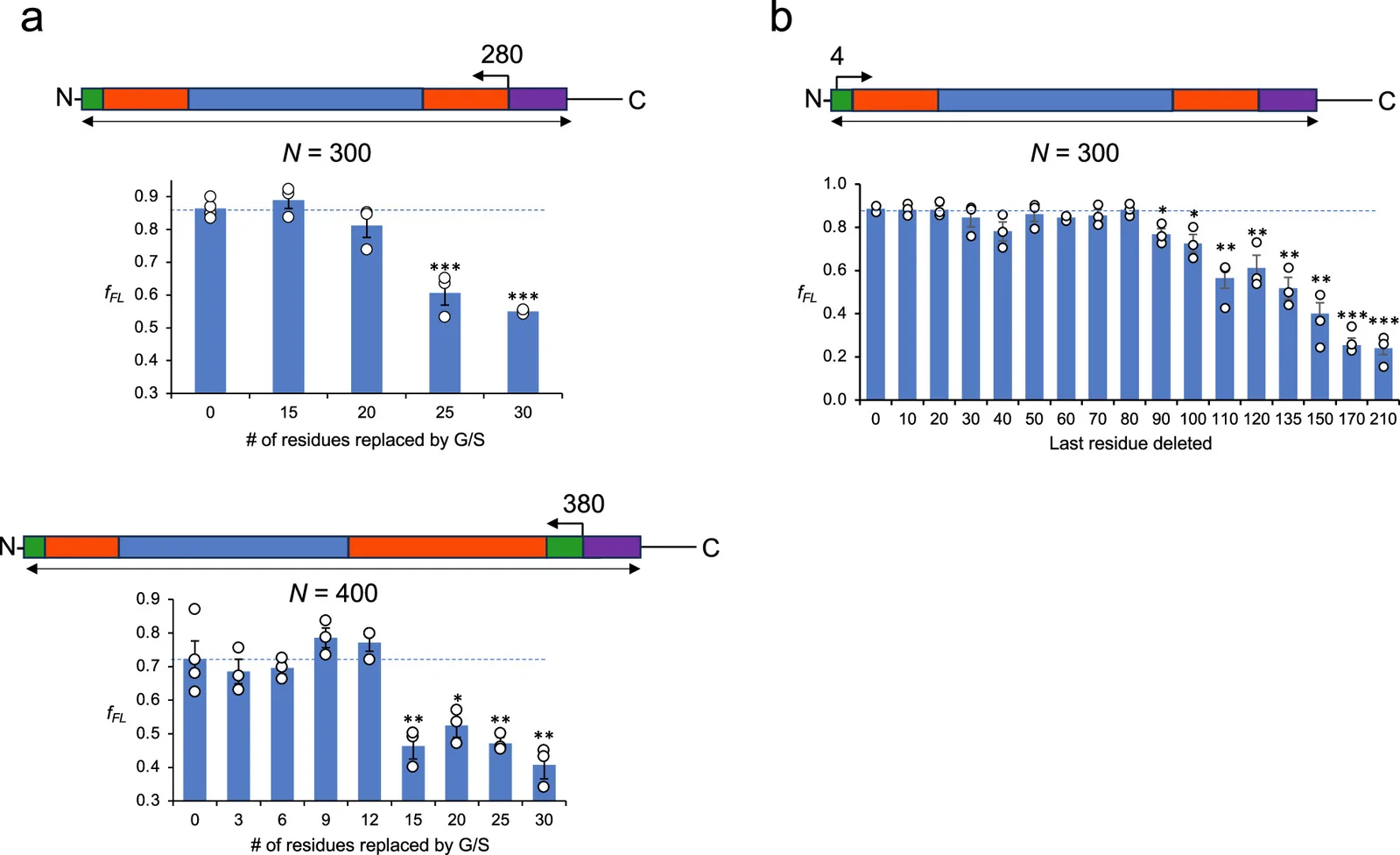
GS- and deletion-mapping of the *N* = 300 and *N* = 400 residues peaks in the FLuc force profile. **a** FLuc residues immediately upstream of the 20-residue AP (purple) are progressively replaced in the C-to-N-terminal direction by alternating G and S residues, as indicated by the arrow. For the *N* = 300 residues construct (top), the replacements start at FLuc residue 280, and for the *N* = 400 residues construct (bottom) at FLuc residue 380. GS-mapping results for the *N* = 300 and *N* = 400 residues peaks in the FLuc FP are shown below. Individual data points are shown as white circles, and blue bars represent averages for *n* ≥ 3 biological replicates, with the exact *n* values given in the Source Data file. Significant differences between the *f*_*FL*_ values of the non-mutated and mutated constructs are indicated (*p* < 0.05 (*), *p* < 0.01 (**), *p* < 0.001 (***); calculated by a two-sided Student’s *t* test). Error bars indicate the standard error of the mean (SEM). **b** N-terminal residues (starting at FLuc residue Ala^4^) are progressively deleted in the N-to-C-terminal direction, as indicated by the arrow. N-terminal deletion results for the *N* = 300 residues peak in the FLuc FP are shown below. Individual data points are shown as white circles and blue bars represent averages for *n* ≥ 3 biological replicates, with the exact *n* values given in the Source Data file. Significant differences between the *f*_*FL*_ values of the original and the deletion constructs are indicated (*p* < 0.05 (*), *p* < 0.01 (**), *p* < 0.001 (***); calculated by a two-sided Student’s *t* test). Error bars indicate the standard error of the mean (SEM). Source data are provided in the Source Data file.

**Fig. 5:**
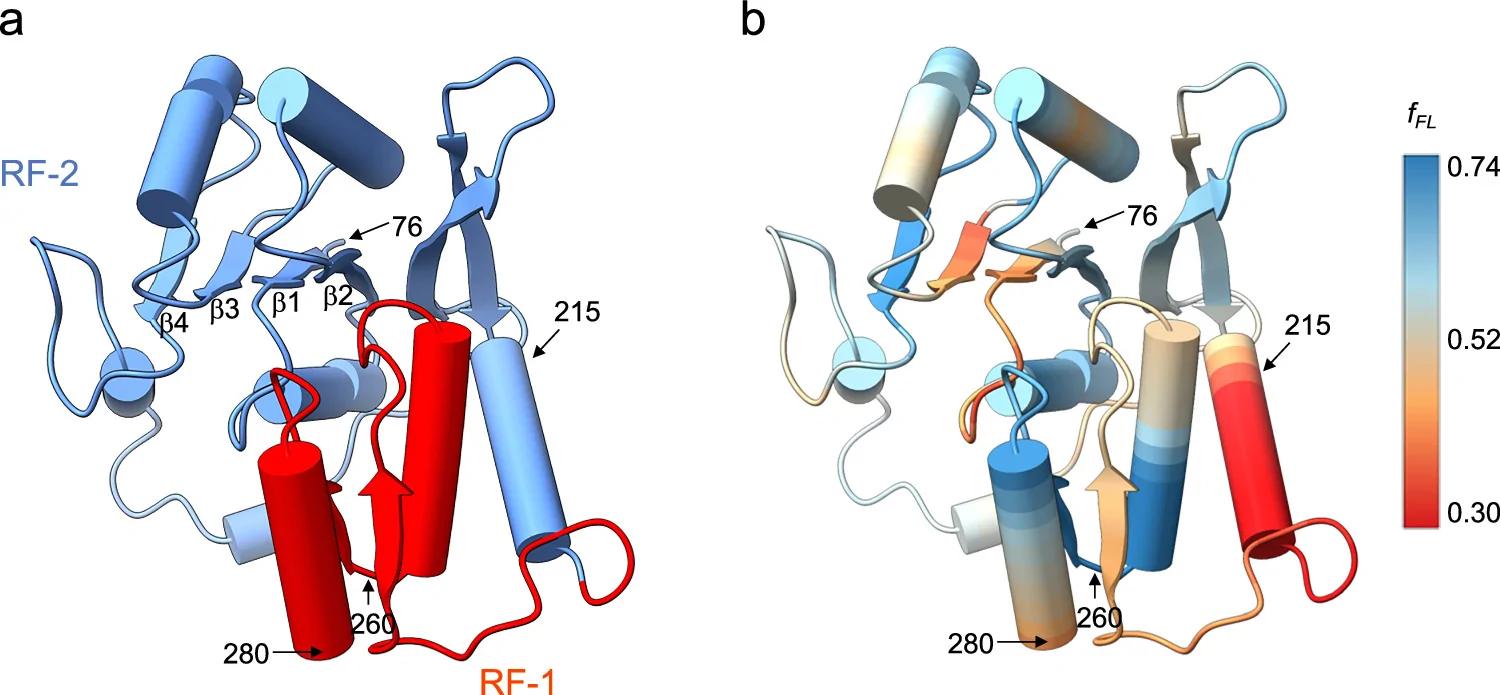
FLuc residues 76-280. **a** Colored by domain structure, c.f., Fig. 1. **b** Colored according to *f*_*FL*_ values (low (0.30) to high (0.74) red-yellow-blue spectrum). The coloring of a given residue represents the instantaneous pulling force on the AP at the point when this residue is located 40 residues away from the PTC. Thus, the dip in the FP at *N* = 255–265 residues (Fig. 3) corresponds to the emergence of the α-helix starting at residue 215 (red), and the peak in the FP at *N* ≈ 300 residues corresponds to the emergence of the α-helix ending at *N* = 260 residues (deep blue), from the ET.

A very strong pulling force is apparent at *N* = 295–310 residues, signaling a cooperative folding event. This peak has the highest amplitude of all. To precisely map its origin, we made a “GS-scan”^42^ where we replaced an increasing number of residues at the C-terminal end of the FLuc part in the *N* = 300 construct by a repeating Gly-Ser sequence. As seen in Fig. 4a, the amplitude of the peak is reduced when ~20 or more C-terminal FLuc residues are replaced by an equally long GS-stretch. Because the SecM(*Ec*) AP part is 20 residues long (Fig. 2a), this means that the C-terminal end of the part of FLuc that folds in this construct is ~40 residues distant from the PTC, as anticipated. In addition, a series of N-terminal deletions in the *N* = 300 residues construct shows that residues 4–80 can be deleted with no effect on the amplitude of the *N* = 300 peak, while longer deletions ending at residues 90 to 170 lead to a gradual reduction in peak amplitude, Fig. 4b. The peak at *N* = 295–310 residues is thus generated by the folding of residues ~80 to ~260, and the potential folding intermediate corresponding to the dip at *N* = 320 residues neatly corresponds to the FLuc RF-2 domain (except for the last, non-contiguous RF-2 segment M^396^-K^414^, Fig. 1) and a small part of RF-1 (most notably the hydrophobic helix G^246^-C^258^), Fig. 5. Consistent with this, the coarse-grained structure-based model of cotranslational folding developed by Jacobs & Shakhnovich^44,45^ predicts that a first, stable folding intermediate should form when FLuc residues ~260–280 emerge from the ET at *N* ≈ 300 residues (dark green bar in Fig. 3). We cannot tell from the FP-data whether the N-terminal part of RF-1 (residues 15–70) is also folded in the *N* = 320 construct, only that it does not contribute to the generation of the *N* = 300 peak.

The gradual reduction in the peak amplitude seen for the progressively longer N-terminal deletions ending between residues 90 and 170 (Fig. 4b), corresponding to the consecutive removal of RF-2 strands β1, β2, β3, and β4 (see Fig. 5), is puzzling at first sight. However, full-atom MD simulations show that the Δ4-80 fragment (FLuc residues 1-282 with residues 4–80 deleted, see “Methods”) is quite stable during a 1 μs simulation, with the six-stranded β-sheet and the central core around the helix formed by residues 89–98 remaining intact, Supplementary Fig. 2 and Supplementary Movie 1. The Δ4–110 fragment (lacking β1, the core helix, and β2) adopts conformations in which the central β-sheet is broken into two 2-stranded β-sheets and the central core is extensively repacked because of the missing helix (residues 89–98), Supplementary Movie 2. The RMSD and RMSF values remain small, however, suggesting a fairly stable folded state, Supplementary Fig. 2. In contrast, the Δ4–150 fragment (lacking most of the N-terminal half of the RF-2 domain) adopts rapidly interchanging conformations and is characterized by high RMSD and RMSF values, Supplementary Fig. 2 and Supplementary Movie 3. Thus, the MD results are broadly consistent with the gradual drop in *f*_*FL*_ values seen in Fig. 4b.

The strong peak-dip pattern at *N* = 400–410 residues (Fig. 3) suggests another folding intermediate. GS-mapping of the *N* = 400 residues construct shows that the C-terminal end of the parts that fold is ~35 residues away from the PTC, Fig. 4a, i*.e*., close to the border between the RF-1 domain and the β-roll domain. Indeed, proteinase K treatment of stalled ^1/2^N ribosomes (corresponding to the *N* = 410 construct) yields a ~ 35 kDa protease-resistant N-terminal FLuc fragment and HDX-MS analysis of ^1/2^N RNCs shows that FLuc residues 1 to ~350 mostly adopt native-like structure^22^.

Finally, the deep dip at *N* = 468-472 residues corresponds to the full emergence of the β-roll domain outside the ET, and matches the predicted folding intermediate at *N* = 455–470 residues, Supplementary Fig. 1. The absence of high peaks in the FP during the exit of the β-roll domain from the ET (*N* ≈ 410–475 residues; FLuc residues ~370–435) suggests that it may not be as stably folded as the other subdomains, in agreement with results obtained by HDX-MS^22^.

Since, given its flexible attachment to the NTD, it seemed likely that the CTD can fold by itself, we recorded the last part of the FP both for full-length FLuc and for a FLuc segment (residues 423–550) encompassing the isolated CTD. Notably, while the two FPs are identical within experimental error between *N* = 500–555 residues, the isolated CTD FP consistently has significantly higher *f*_*FL*_ values (by about 0.2, on average) between *N* = 560–605 residues, while the shapes of the two FPs remain very similar, Supplementary Fig. 3a. Thus, the presence of the NTD causes a significant destabilization of the second half of the CTD in the ribosome-bound NC, again in agreement with HDX-MS data^22^.

### Cryo-EM analysis of FLuc RNCs

**Fig. 6:**
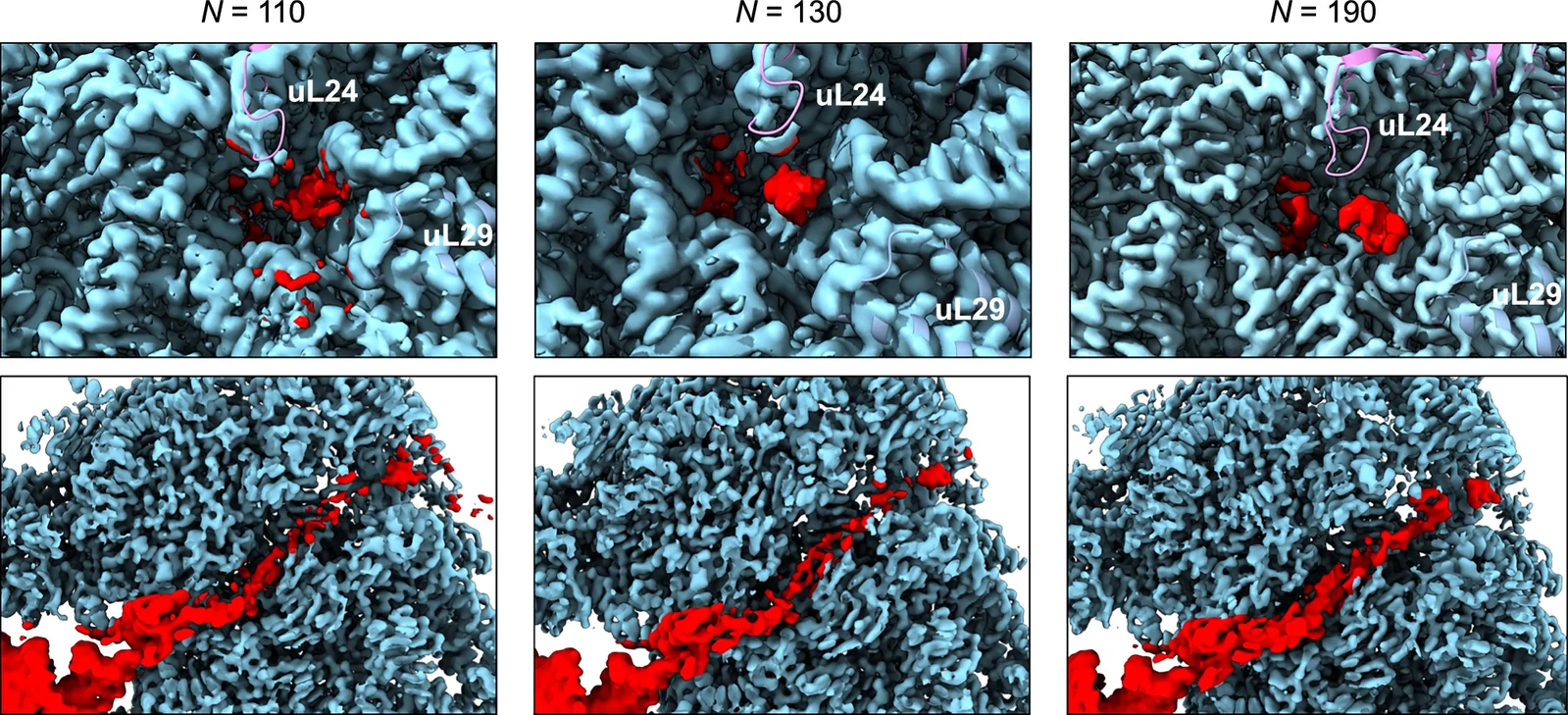
Cryo-EM densities of E. coli ribosomes with FLuc constructs *N* = 110, 130, and 190 residues stalled in the exit tunnel. Composite maps are shown with NC-density in the exit tunnel colored red. Ribosomal proteins uL24 and uL29 from PDB 3JBU (rigid body-docked) are shown in ribbon representation. Top panels: the ribosome exit port area, bottom panels: cross-section showing the NC-density (red) in the ET (part of the tRNA density is seen in the lower left corner).

To ascertain whether early peaks in the FP might reflect the formation of small folded subdomains inside the ET, as has been seen for stably folded proteins up to ~100 residues in size^11^, we picked three constructs corresponding to the peaks at *N* = 110, 130, and 190 residues (Fig. 3) for analysis by cryo-EM. RNCs carrying these constructs stalled by the strong SecM(3 W) AP^37^ were purified from *E. coli* cells^46^, and structures were determined at an average resolution of 2.3 Å. For all three constructs, stalled NCs were readily apparent in the ET but with only small compact densities evident near the uL24 loop in the exit port, Fig. 6. The absence of more extensive folded domains in the ET shows that these early FP peaks are not generated by the formation of stable substructures within the ET, but rather represent compaction of the NC outside the ribosome. While we cannot completely rule out the presence of highly mobile, folded substructures within the vestibule, we consider this unlikely, as even small folded domains tend to fill up the available space in the ET^3^ leaving little room for movement.

### Effects of TF and TF[2xRBD] on the FLuc FP

In *E. coli*, Trigger Factor (TF) is the first chaperone that meets the NC as it emerges from the exit port^47,48^. NC-TF interactions can typically be detected when the NC is 80–120 residues long and begins to form partially folded conformers^48,49^. A host of studies have shown that TF preferentially binds compacted/partially folded substrates in a wide cavity located ~50–100 Å away from the ribosome exit port^7,19,23,50–52^. FP analysis of the cotranslational folding of DHFR has shown that TF reduces the amplitude of the peak that represents the main folding event^53^, suggesting that TF binding to partially folded states may compete with formation of the fully folded structure. Given the multiple compaction/folding events apparent in the FLuc FP, we tested the effects of TF and of a TF[2xRBD] variant, in which duplication of the ribosome-binding domain positions the substrate-binding cavity farther from the exit port than in wild-type TF^7^.

**Fig. 7:**
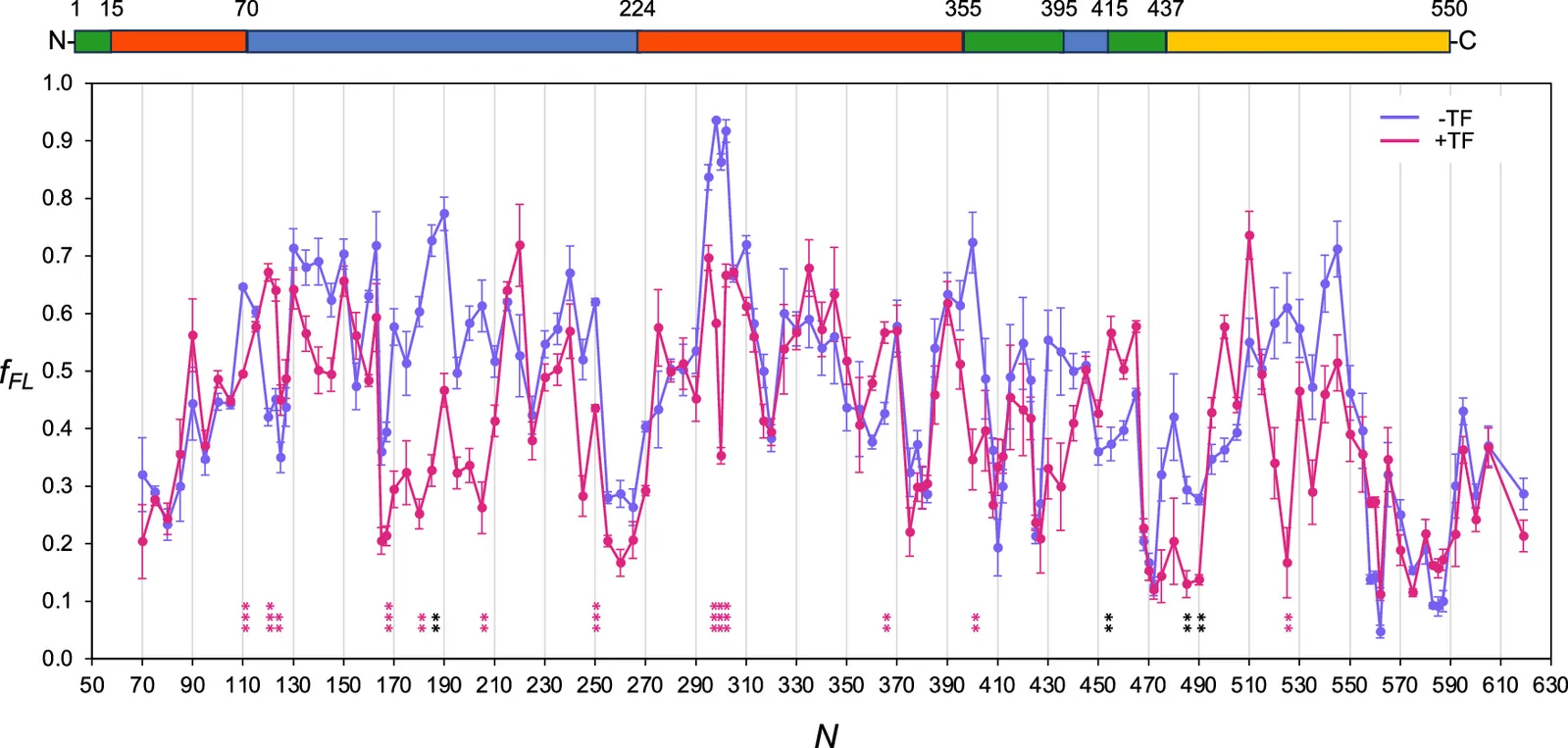
Effects of trigger factor on the FLuc force profile. FLuc FP obtained in the presence of 4 μM TF (dark red), superimposed on the original FP from Fig. 3 (blue). All data points represent averages for *n* ≥ 3 biological replicates, with the exact *n* values given in the Source Data file. Constructs for which the difference between the ±TF *f*_*FL*_ values are significant on the *p* < 0.01 (**) or *p* < 0.001 (***) levels (calculated by a two-sided Student’s *t* test) are indicated. When marked in red, the differences are also significant on the *p* < 0.05 level according to the Holm-Bonferroni method^70^, see “Methods”. Error bars indicate the standard error of the mean (SEM). Source data are provided in the Source Data file.

The FLuc FP obtained in the presence of TF, Fig. 7 (red curve), is overall quite similar to the original FP but with some interesting differences. The most dramatic difference is for the ~40-residue long region *N* = 165–205 residues, where the *f*_*FL*_ values are markedly reduced (by about 0.25, on average) in the presence of TF, even though the shapes of the two FPs remain similar, Supplementary Fig. 3b. Significant but more localized reductions in *f*_*FL*_ are seen at *N* ≈ 110, 250, 300, 400, and 525 residues, and significant increases in *f*_*FL*_ at *N* ≈ 125 and 365 residues (the apparent reduction at *N* = 135–145 is not statistically significant on the *p* < 0.01 level). Similar patterns of localized effects on the FP by TF have been seen for a couple of other multi-domain proteins^4^.

The partial FP recorded in the presence of TF[2xRBD] (which we focused on areas where there are significant TF effects), Supplementary Fig. 4, is even more similar to the original FP, and the dramatic TF effects seen at *N* = 165–205 are not seen with TF[2xRBD]. Notably, however, the amplitudes of the main peaks at *N* = 300, 400, and 525 residues are markedly reduced by TF[2xRBD], albeit the effects are smaller than seen with TF.

Interpretation of the FLuc-TF and FLuc-TF[2xRBD] FPs is complicated by the fact that the extended TF binding cavity is located far from the ribosome exit port, where the compaction/folding events that are reflected in the FP presumably occur. Depending on where a particular substrate domain binds in the binding cavity, a minimum of ~15–20 NC residues in an extended conformation would be required to reach from the exit port to the binding cavity^54,55^. Indeed, crosslinking analysis of interactions between folding intermediates in *E. coli* β-galactosidase NCs and TF shows that the number of NC residues between the PTC and the most C-terminal NC residue found to crosslink to TF typically ranges from ~80 to ~150 residues, but can be as short as ~60 residues^7^, in agreement with ribosome profiling data^49^.

Thus, the *f*_*FL*_ value recorded at a given *N*-value in the presence of TF should reflect the combined effects of a TF-binding event happening at least ~20 residues away from the exit port and simultaneous folding/compaction events taking place at the exit port. Previous fluorescence-based studies have shown that a ribosome-stalled FLuc translation intermediate of length 77 residues (comparable to our *N* = 77 construct) does not interact with TF, while a translation intermediate of length 164 residues (comparable to our *N* = 164 construct) both recruits TF to the ribosome and interacts with the binding cavity, albeit considerably less efficiently than a long, 520-residue intermediate^56^. This fits nicely with the TF-induced reduction in *f*_*FL*_ values starting at *N* ≈ 165 residues, strongly suggesting that binding in the TF cavity competes with (but, because the FP shape stays the same, does not completely eliminate) compaction/folding at the exit port between *N* ≈ 165–210 residues.

Assuming that 40 residues are sequestered in the ET, a possible interpretation of the early parts of the +TF FP is that compaction at the exit port commences no later than when ~90 residues have emerged from the ET (*N* ≈ 130 residues), that the compacting NC can reach the TF-binding cavity when ~125 residues have emerged from the ET (*N* = 165 residues) at which point TF-binding starts to compete with continued compaction at the exit port (thus reducing *f*_*FL*_), and that formation of a more stable compacted state at the exit port then outcompetes TF-binding, starting at *N* ≈ 210 residues. The compacted part increases in size until the first main compacted intermediate, encompassing residues ~1–210, is complete at *N* ≈ 250 residues, Fig. 7. Given that TF binds compacted/partially folded rather than unfolded regions in a NC^7,48,57^, the TF-induced reduction in *f*_*FL*_ values starting at *N* = 165 residues is a strong indication that the FLuc NC forms a compacted intermediate no later than when ~125 residues have emerged from the ET and that the intermediate-amplitude region in the -TF FP from *N* = 130–250 residues reflects a continuous compaction/folding process, rather than an abrupt, cooperative folding event. A similar scenario might apply for the *N* ≈ 520-545 residues region in the CTD.

The strong, narrowly localized reductions in *f*_*FL*_ caused by TF at the maxima of the major peaks at *N* ≈ 300 and 400 residues are also seen with TF[2xRBD], which is surprising since the binding cavity is at different distances away from the exit port in TF and TF[2xRBD]. A possible way out of this conundrum is if these TF effects are mainly caused by interactions between the NC and the TF RBD rather than the central TF cavity; some evidence for the existence of a NC binding site in the RBD has recently been obtained by chemical crosslinking^7^.

## Discussion

Cotranslational folding of multi-domain proteins has so far been studied mainly by biophysical methods such as NMR^6^, HDX-MS^22^, and optical tweezers^58^. Data obtained with these kinds of methods often have rather straight-forward structural interpretations, but are laborious to obtain and hence normally derived from the analysis of a limited number of stalled ribosome-nascent chain complexes (RNCs) for any given target protein.

Force-profile analysis (FPA) allows more fine-grained study of cotranslational folding—in principle with up to single-residue resolution—but has not yet been applied to multi-domain proteins, except in a high-throughput fluorescence-based in vivo format^4^. Here, we have applied the “classical”, gel-based FPA method^3,34^ to map the cotranslational folding of the 550-residue multi-domain protein FLuc at five-residue resolution, both in the absence and presence of the TF chaperone. FLuc has an interesting architecture, with a relatively large NTD flexibly attached to a relatively small CTD. The NTD, in turn, is composed of two intertwined Rossmann folds and a β-roll domain, and it has been unclear to what extent identifiable folding intermediates, in addition to the semi-independent NTD and CTD, can form during translation.

Overall, the FLuc FP (Fig. 3) is characterized by long regions of fluctuating *f*_*FL*_ values of intermediate amplitude, punctuated by one major peak at N ≈ 300 residues and a few deep dips that we propose represent the formation of transient folding intermediates.

The early part of the FP, up to *N* = 250 residues, shows an intricate pattern of medium-amplitude peaks and dips, quite distinct from the characteristic high-amplitude, sharp peaks previously seen in FPs for both single- and multi-domain proteins^3,4,11,18,20,39^. This region also shows clear signs of TF-binding (Fig. 7), suggesting the formation of a compacted/partially folded NC. This is in agreement with HDX-MS data for the ^1/2^N_s_ and N_s_ RNCs (corresponding to our *N* = 145 and *N* = 230 constructs)^22^, and with the general observation that on-ribosome folding promotes formation of partially folded intermediates^13^.

As shown by N-terminal deletion and C-terminal GS-replacement analysis, the main peak at *N* ≈ 300 residues signals a cooperative folding event involving FLuc residues ~80–260, precisely matching the RF-2 domain plus an additional helix located at the border between the RF-2 domain and the second half of the RF-1 domain (Fig. 5). Interestingly, deletion of the first half of the RF-1 domain has no effect on the amplitude of the *N* ≈ 300 peak. In itself, this part of the RF-1 domain therefore does not seem to contribute much to the stability of the RF-2 domain.

The next strong peak-dip pattern, at *N* = 400–410 residues, pinpoints the co-translational formation of a stable RF-1/RF-2 folding intermediate, in agreement with HDX-MS data for the ^1/2^N construct^22^. Finally, the deep dip at *N* ≈ 470 residues matches the predicted folding intermediate at *N* = 455–470 residues and represents the folding of the full NTD. The CTD folds largely independently of the NTD, albeit its overall stability appears to be somewhat reduced by the presence of the latter.

The intermediate amplitude regions in the FP imply an almost continuous series of small compaction/folding events during chain elongation. The absence of high peaks and deep dips in these regions suggests that these events are non-cooperative, possibly reflecting ongoing compaction and/or the addition of small pieces of structure to a more or less stably folded core, each piece providing only a marginal contribution to the overall stability. Electrostatic interactions with the negatively charged ribosome surface might also contribute^8,59^.

The effect of TF on the FP is broadly consistent with this picture of the cotranslational folding process, especially in the region *N* = 165–210 residues. The large reduction in *f*_*FL*_ over this region implies a strong interaction with TF, in turn implying that the NC is compacted/partially folded (as also shown by the HDX-MS data^22^ for the N_S_ construct). Nevertheless, for *N* ≥ 215 residues, compaction at the exit port apparently outcompetes TF-binding. A similar pattern is seen for the CTD, where TF markedly reduces *f*_*FL*_ for the region *N* = 520–545 residues, presumably by binding to the early parts of the CTD and thereby preventing folding at the exit port. In addition, the FP data have uncovered narrowly localized effects of TF on two of the main peaks in the FP, at *N* = 300 and 400 residues. These effects are also seen with TF[2xRBD], hinting at a possible involvement of the TF RBD.

While our analysis here has focused on the main peaks and dips in the FLuc FP, we surmise that the intricate medium-amplitude features seen throughout the FP also hold important information on the compaction and folding of the growing NC. Single-residue resolution FPA studies, together with mutational analysis and computational modeling, may eventually make it possible to gain a detailed, residue-level understanding of both the initial compaction/partial folding processes evident in the first half of the FLuc FP and the later growth of the folded domains in the second half. Promising steps in this direction have already been taken for the small single-domain protein HemK-NTD^60^.

## Methods

### Enzymes and chemicals

Cloning enzymes, reagents, and buffers were acquired from New England BioLabs (Ipswich, Massachusetts, USA) and Thermo Fisher Scientific (Waltham, Massachusetts, USA). Oligonucleotides were acquired from Eurofins (Germany). [^35^S] L-Methionine was acquired from Revvity (Waltham, MA, USA). All other chemicals were acquired from Sigma-Aldrich (subsidiary of Merck Life Science, Darmstadt, Germany) unless otherwise stated.

### Purification of E. coli trigger factor

TF was expressed from the plasmid pCA528^61,62^. containing an Ulp1-cleavable N-terminal His_6_-SUMO tag. The plasmid was transformed into *E. coli* BL21(DE3)-T1R competent cells. Terrific Broth growth media (TB, modified, Sigma-Aldrich) supplemented with kanamycin (100 µg/ml) and glucose (0.4%) was inoculated with a fresh transformant and grown in a shaking incubator overnight at 30 °C. The starter culture was then used to inoculate 1500 ml TB supplemented with 1% glycerol and kanamycin (50 μg/ml). The culture was grown at 37 °C until an OD_600_ of 2 was reached, when the temperature was lowered to 18 °C. Protein expression was induced by the addition of IPTG (Thermo Fisher Scientific, Waltham, MA, USA) to a final concentration of 0.5 mM and expression was continued for 16–20 h. Cells were harvested by centrifugation (10 min at 4500 × *g*, 4 °C), re-suspended in lysis buffer (50 mM HEPES, 150 mM KCl, 5% glycerol, pH 7.5) supplemented with nuclease (own production) and cOmplete EDTA-free protease inhibitor cocktail (Roche, Basel, Switzerland). The resuspended cells were frozen and stored at -80 °C.

The resuspended cells were thawed and disrupted by sonication (4 s/4 s 4 min, 80% amplitude, Sonics Vibracell-VCX750, Sonics & Materials Inc., Newtown, CT, USA). Lysates were centrifuged at 49,000 × *g* for 20 min at 4 °C. The supernatants were filtered (Nalgene™ Rapid-Flow™ Bottle top filters, 0.45 µm, Thermo Fisher Scientific, Waltham, MA, USA) before loading onto an IMAC HisTrap HP 5 ml column (Cytiva, Little Chalfont, UK), mounted on an ÄKTA Xpress FPLC system (Cytiva, Little Chalfont, UK). After washing with wash buffer (25 mM HEPES, 500 mM KCl, 5% glycerol, pH 7.5), the protein was eluted with elution buffer (25 mM HEPES, 150 mM KCl, 5% glycerol, 250 mM imidazole, pH 7.5), and further purified by size exclusion chromatography using a HiLoad 16/60 Superdex 200 preparative grade column (Cytiva, Little Chalfont, UK) pre-equilibrated with gel filtration buffer (25 mM HEPES, 150 mM KCl, 5% glycerol, pH 7.5).

Fractions were examined by SDS-PAGE, and fractions containing the target protein were pooled. The sample was supplemented with 0.5 mM DTT, and SUMO protease Ulp1 was added at a 1:100 molar ratio. The reaction was incubated at 4 °C overnight. Aliquots of the reaction mixture were taken after 16 h and analyzed by SDS-PAGE. Protein expression and purification were carried out by the Protein Science Facility at Karolinska Institutet, Stockholm, Sweden.

### Cloning and mutagenesis

FLuc plasmids were produced by Invitrogen GeneArt Services, Thermo Fisher Scientific. The DNA sequence coding for Firefly (*Photinus pyralis*) Luciferase (UniProt Q27758) is a codon-optimized variant^22^ provided by the lab of David Balchin. The full-length pET19b plasmid contains—after modification—the 550-residue long FLuc sequence, a 49-residue GS-linker sequence, the *E. coli* SecM AP SGSFSTPVWISQAQGIRAGP (note the SGS tripeptide included to “insulate” the AP from FLuc), and a C-terminal tail derived from the *lepB* gene, under the control of a T7 promoter^30,33,34,63^. Stepwise internal deletions upstream of the AP resulted in 109 truncated constructs that differ in length by 5 amino acids. Further mutagenesis was performed in-house by a combination of Q5 PCR and Gibson assembly^64^ with a homemade reaction mixture.

### In vitro transcription and translation

The FLuc-encoding regions, including a 5’ T7 promoter, were individually amplified by PCR from the plasmid set. PCR products were purified and subjected to in vitro transcription and translation according to the PURExpress^TM^ protocol from New England Biolabs. The PURExpress^TM^ kit contains the essential components required for in vitro transcription and translation isolated from *E. coli*. Purified wild-type and mutant Trigger Factor proteins (TF, TF[2xRBD])^7,65^ were prepared at 40 μM in PURExpress system buffer (9 mM magnesium acetate, 5 mM potassium phosphate, pH 7.3, 95 mM potassium glutamate, 5 mM NH4Cl, 1 mM spermidine, 8 mM putrescine, 1 mM DTT) and included in the in vitro reactions at a final concentration of 4 μM. After the addition of 0.8 μL [^35^S]-Methionine, PURExpress reactions were incubated for 15 min at 37 °C with shaking at 700 rpm in an Eppendorf Thermomixer. The reaction was stopped by the addition of 10% ice-cold TCA, and samples were incubated for 30 min on ice, then centrifuged for 5 min at 20,800 × *g* at 4 °C. The pellets were dissolved in sample buffer (67 mM Tris, 3.3% (w/v) SDS, 0.012% (w/v) bromophenol blue, 10 mM EDTA-KOH pH 8.0, 6.75% (v/v) glycerol, and 100 mM DTT) and treated with 0.25 mg/mL RNase at 37 ^o^C for 15 min. The mixture was then separated by SDS/PAGE, and the radiolabeled proteins were detected using a Fuji FLA-3000 scanner.

### Quantification

Full-length and arrested band intensity profiles were extracted with ImageJ^66^. The profiles were then fit to two or more Gaussian curves with our in-house software EasyQuant (https://github.com/gvh-lab/easyquant)^67^ to determine the integrated band intensities, Supplementary Fig. 5.  *f*_FL_ was calculated as *f*_*FL*_ = *I*_*FL*_/(*I*_*FL*_ + *I*_*A*_), where *I*_*A*_ and *I*_*FL*_ are the integrated intensities of the arrested and full-length bands, respectively; all measured *f*_FL_ values are reported in the Source Data file. For certain constructs where band assignment was ambiguous, control constructs were cloned and expressed. Full length-size control constructs were made by mutating the C-terminal Pro in SecM(*Ec*) to Ala, which prevents SecM(*Ec*)-induced stalling. Arrested-size controls were made by mutating the C-terminal Pro in the SecM(*Ec*) to a stop codon.

### Coarse-grained cotranslational folding model

To predict cotranslational folding intermediates, we used the Jacobs & Shakhnovich method^45^. Briefly, this method takes one or more native PDB structures and sequence information as input and performs a mean-field theory calculation to determine the minimum free energy (in units of k_B_T) of a nascent chain as the nascent chain length, *L*, increases, starting from the N-terminus. Multiple PDB structures are used as input so only consensus contacts, those present in all structures, are counted. The minimum free energy decreases when the energetically favorable native contacts outweigh the entropy cost of an unfolded nascent chain of the same length. We used the full-length FLuc sequence (Supplementary Data 1) and two Firefly Luciferase PDB structures as input: a 2.4 Å apo crystal structure^68^ (PDB 5DV9), and a 2.999 Å crystal structure in the presence of an inhibitor^69^ (PDB 5WYS). The RMSD between the two is 1.033 Å. Supplementary Fig. 1 was produced with the software available at https://shakhnovich.faculty.chemistry.harvard.edu/software/coarse-grained-co-translational-folding-analysis according to the instructions contained therein, using PDB structures 5DV9 and 5WYS as input.

### Statistics

To correct for multiple testing and correlations in the FP when comparing the FLuc[±TF] FPs (Fig. 7), the autocorrelation function (ACF) was first calculated for the FLuc[-TF] FP (109 data points; only data points for *N-*values that are multiples of 5 were included). The ACF showed significant values for lags 1–3 (*i.e*., for residue separations up to 15 residues), Supplementary Fig. 6. The Holm-Bonferroni method^70^ was then used to identify the *N*-values for which the FLuc[±TF] *f*_*FL*_ values differed on the α = 0.05 level, with *p*-values calculated by Student’s *t* test and the number of independent tests set to *m* = 109/4 = 27.

### Molecular dynamics simulations

Three models, Δ4-80, Δ4-110, and Δ4-150, were created based on the AlphaFold DB^71^ model AF-P08659-F1-v4. Each model structure was trimmed as indicated by the model name. For all three models, the residues at the N-terminus were mutated using PyMOL^72^ to add the “MED” sequence. The terminal residues were capped with ACE (N-acetyl) and NME (N-methylamide). The sequences of the constructs are shown below:

Δ4-80:

MEDCSENSLQFFMPVLGALFIGVAVAPANDIYNERELLNSMGISQPTVVFVSKKGLQKILNVQKKLPIIQKIIIMDSKTDYQGFQSMYTFVTSHLPPGFNEYDFVPESFDRDKTIALIMNSSGSTGLPKGVALPHRTACVRFSHARDPIFGNQIIPDTAILSVVPFHHGFGMFTTLGYLICGFRVVLMYRFEEELFLRSLQDY

Δ4-110:

MEDERELLNSMGISQPTVVFVSKKGLQKILNVQKKLPIIQKIIIMDSKTDYQGFQSMYTFVTSHLPPGFNEYDFVPESFDRDKTIALIMNSSGSTGLPKGVALPHRTACVRFSHARDPIFGNQIIPDTAILSVVPFHHGFGMFTTLGYLICGFRVVLMYRFEEELFLRSLQDY

Δ4-150:

MEDIMDSKTDYQGFQSMYTFVTSHLPPGFNEYDFVPESFDRDKTIALIMNSSGSTGLPKGVALPHRTACVRFSHARDPIFGNQIIPDTAILSVVPFHHGFGMFTTLGYLICGFRVVLMYRFEEELFLRSLQDY

The all-atom systems were prepared with GROMACS 2024.4^73^. Each protein was solvated in a rhombic dodecahedral box of explicit TIP3P^74^ water, with a minimum distance of 1.5 nm between the solute and the edge of the box. The systems were neutralized with the minimum number of Na⁺ or Cl⁻ ions required. As we simulated the constructs in the absence of a ribosome, we did not use the same experimental conditions as in the Force Profile Analysis. Supplementary Table 1 summarizes the composition of each system, including box dimensions and total atom counts.

Molecular dynamics simulations were performed using the Amber99SB-ILDN force field^75^ with GROMACS 2024.4^73^. Enhanced sampling methods were not used because the objective of the MD simulations was to assess whether the constructs were stable in the absence of external forces, within a 1 µs timescale. We did not intend to characterize the full conformational landscape of these constructs; therefore, enhanced sampling methods were not employed.

For each system (Δ4–80, Δ4–110, and Δ4–150), 3 independent replicas were generated. The systems were energy minimized using steepest descent, then the equilibration protocol described below was followed:

0–500 ps: NVT and position restraints on all heavy atoms (force constant of 1000 kJ mol^−1^nm^−2^), temperature coupling to 310 K using V-rescale thermostat^76^.

500ps–1 ns: NPT and position restraints on all heavy atoms (force constant of 1000 kJ mol^−1^nm^−2^), with a Berendsen barostat^77^ with a coupling constant τp = 0.5 ps and an isotropic compressibility of 4.5·10^−5 ^bar^−1^, temperature coupling to 310 K using V-rescale thermostat.

For each replica, a 1 µs production simulation was performed in the NPT ensemble with periodic boundary conditions. Temperature coupling was done with the Nose-Hoover thermostat (310 K)^78^, and pressure coupling was done with the Parrinello-Rahman barostat (τp = 5 ps and an isotropic compressibility of 4.5·10^−5 ^ bar^−1^)^79^. A 10 Å cut-off was used for van der Waals and short-range electrostatic interactions. The Particle-Mesh Ewald (PME) summation method was used for long-range electrostatic interactions^80^. Verlet cut-off scheme was used^81^. Covalent bonds were constrained using the LINCS algorithm^82^. The integration time step was 2 fs for all steps.

Trajectories were filtered by extracting a frame every 100 ps. We discarded the first 100 ns to guarantee that the system had equilibrated before analysis, Supplementary Fig. 7.

The root mean square fluctuation (RMSF) and root mean square deviation (RMSD) relative to an average conformation for the simulation period between 0.1 and 1 µs were calculated, as described below:

The RMSF of the C_α_ atoms of each frame in the filtered trajectories, relative to the average structure, was computed using the “gmx rmsf” function in GROMACS. The average conformation was calculated from the sampled conformations between 0.1 and 1 µs for each trajectory. The average structure for each trajectory was saved as a PDB file, and the mean RMSF value and standard deviation for each C_a_ atom were calculated for each system. The root mean squared distance (RMSD) of the C_α_ atoms to the average structure (obtained during the RMSF calculation) was calculated using the “gmx rms” function in GROMACS and fitting the C_α_ atoms for both rotation and translation (-fit rot+trans). RMSD to the initial conformation after energy minimization was calculated in the same way. To create the RMSD plots, the “histplot” function in seaborn^83^ was used to compute distributions of RMSD values from the three trajectories for each system.

All plots were generated in Python 3.10 with matplotlib^84^ and seaborn^83^. Structure images were created using ChimeraX^85^. Movies were generated using VMD^86^. The initial structures, simulation input files, and trajectories are available in Zenodo (https://zenodo.org/records/20836784). Supplementary movies 1–3 of the full trajectories are available in Zenodo (https://zenodo.org/records/17981108).

### Cryo-electron microscopy

Constructs from in vitro transcription and translation experiments were modified for cryo-electron microscopy (cryo-EM). First, the SecM(3 W) AP (FSTPVWIWWWPPIRGSP) was used to enhance stalling^37^. Second, a 6xHis tag was added to the N-terminus of FLuc to facilitate purification of RNCs (full sequences in Supplementary Data 1). Specifically, we chose three constructs at *N* = 110, 130, and 190 residues. RNCs were purified according to^46^, and expression from pET19b was induced with 1 mM IPTG in *E.coli* BL21(DE3). Purified RNCs were added to grids using a Vitrobot (Thermo Fisher Scientific, Waltham, Massachusetts, USA), plunge-freezing with a 3 sec blot time and 15 sec wait time in 100% humidity at 4 °C. Data were collected on a Krios G3i microscope (Thermo Fisher Scientific, Waltham, Massachusetts, USA) equipped with a Gatan K3 DED at 300 kV with a pixel size of 0.825 Å/pixel, over 40 frames with a total dose of 40 e^–^/Å^2^, Supplementary Table 2. Processing was performed using CryoSPARC 4.3.0. Briefly, after motion correction and CTF estimation, particles were picked using either the built-in “blob” picker or based on templates generated from the data, and extracted with a 550 pixel box size Fourier cropped (“binned”) to 128 pixels. After 2D classification, selected particles were re-extracted unbinned and subjected to 3D homogeneous refinement. Refined particles were then subtracted such that the only density remaining was of the ribosomal groove containing the tRNA. Then, particles were 3D classified based on the tRNA conformation. Finally, particles representing ribosomes with P-site tRNA were selected and refined to generate the final maps. There were slight processing differences between the three constructs. Full workflows are provided in Supplementary Fig. 8. The data was collected at the Swedish National Cryo-EM Facility funded by the Knut and Alice Wallenberg, Family Erling Persson and Kempe Foundations, SciLifeLab, Stockholm University and Umeå University.

### Reporting summary

Further information on research design is available in the Nature Portfolio Reporting Summary linked to this article.

## Supplementary information

Supplementary Information

Description of Additional Supplementary Files

Supplementary Data 1

Supplementary Movie 1

Supplementary Movie 2

Supplementary Movie 3

Reporting Summary

Transparent Peer Review file

## Source data

Source Data

## Data Availability

Cryo-EM maps have been deposited in the Electron Microscopy Data Bank (EMDB) under accession numbers EMD-56368, EMD-56453, and EMD-56462. Initial structures, MD simulation input files, and trajectories are available on Zenodo (https://zenodo.org/records/20836784). PDB codes of previously published structures used in this study are 5DV9, 5WYS and 3JBU. Supplementary Movies 1-3 of the full trajectories are available with this paper and on Zenodo (https://zenodo.org/records/17981108). Supplementary Data 1 contains the amino acid sequences of all constructs used in this work. Source Data are provided as a Source Data file. Source data are provided with this paper.

## Code availability

EasyQuant is available on GitHub (https://github.com/gvh-lab/easyquant) or as an archived release on Zenodo (https://doi.org/10.5281/zenodo.21872778)^67^.
