# Supplementary material for: Quasi-continuous cotranslational compaction and folding of a multidomain protein
Source: Nat Commun. 2026 Oct 5;17:10495. doi: 10.1038/s41467-026-78090-y (PMC13639035; doi:10.1038/s41467-026-78090-y)
Supplement: Supplementary file 1 [file 41467_2026_78090_MOESM1_ESM.pdf]

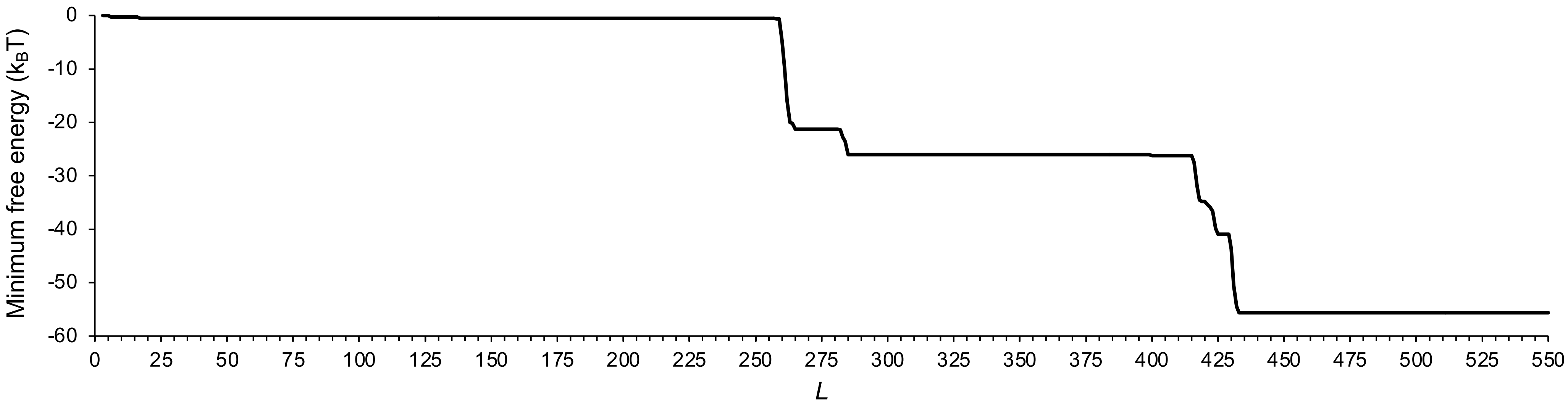

Supplementary Figure 1. Cotranslational folding intermediates predicted by the Jacobs & Shakhnovich method[44]. Folding intermediates are predicted to form at FLuc lengths *L* ≈ 260-285 and 415-430 residues, corresponding to *N* ≈ 300-325 and 455-470 residues (Figure 3). Source data are provided in the Source Data file.

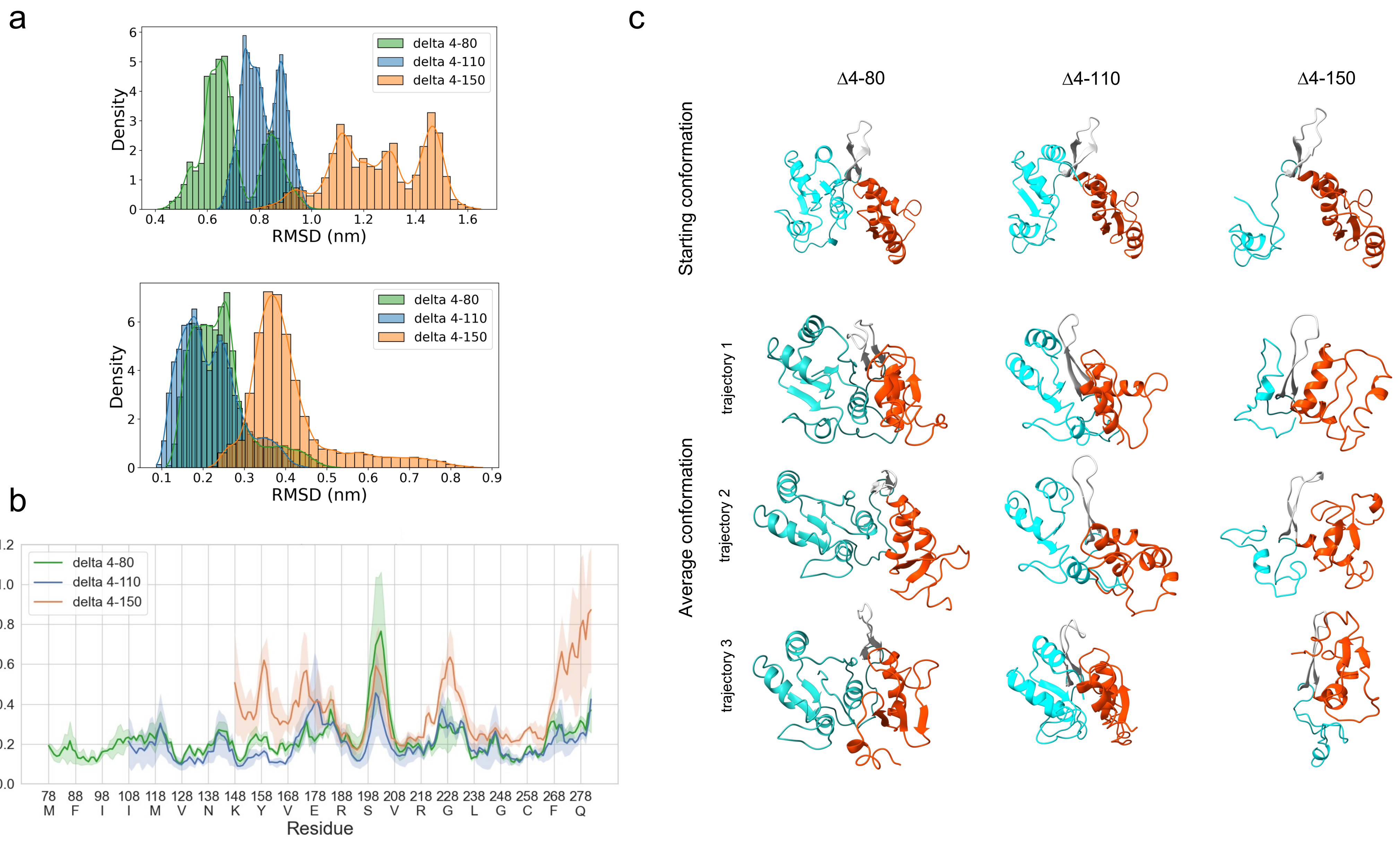

a
delta 4-80
delta 4-110
delta 4-150
Density
RMSD (nm)
b
RMSF (nm)
Residue
78 88 98 108 118 128 138 148 158 168 178 188 198 208 218 228 238 248 258 268 278
M F I I M V N K Y V E R S V R G L G C F Q
c
Δ4-80
Δ4-110
Δ4-150
Starting conformation
Average conformation
trajectory 1
trajectory 2
trajectory 3

continued

Supplementary Figure 2. Full-atom molecular dynamics simulations of FLuc residues 1-282, with residues 4-80, 4-110, and 4-150 deleted. Three 1 μs simulations were performed for each of the three FLuc fragments. (a) RMSD distributions for the Δ4-80 (green), Δ4-110 (blue), and Δ4-150 (orange) FLuc fragments. RMSD values were calculated for each 100 ps simulation frame in the interval 0.1-1 μs using either the starting conformation (panel *c*, top row) or the average conformation of all frames in the same interval (panel *c*, three bottom rows) as the reference. The results for the three simulations for each fragment were pooled. (b) RMSF profiles for the Δ4-80 (green), Δ4-110 (blue), and Δ4-150 (orange) FLuc fragments. C_α RMSF values were calculated for each 100 ps simulation frame in the interval 0.1-1 μs using the average conformation of all frames in the same interval as the reference, and the results for the three simulations performed for each FLuc fragment were averaged (solid line). The shaded area indicates the standard deviation. (c) Starting conformation (initial models after energy minimization, c.f., Figure 5) and the three average conformations (calculated over the interval 0.1-1 μs) for the Δ4-80, Δ4-110, and Δ4-150 FLuc simulations. Structures are colored as in Supplementary Movies 1-3 (N-terminal part in cyan, β-hairpin residues 192-211 in gray, C-terminal part in red). Starting conformations were obtained by deleting the relevant residues from the complete FLuc structure (AF-P08659-F1-model_v4), followed by a steepest-descent energy minimization step.

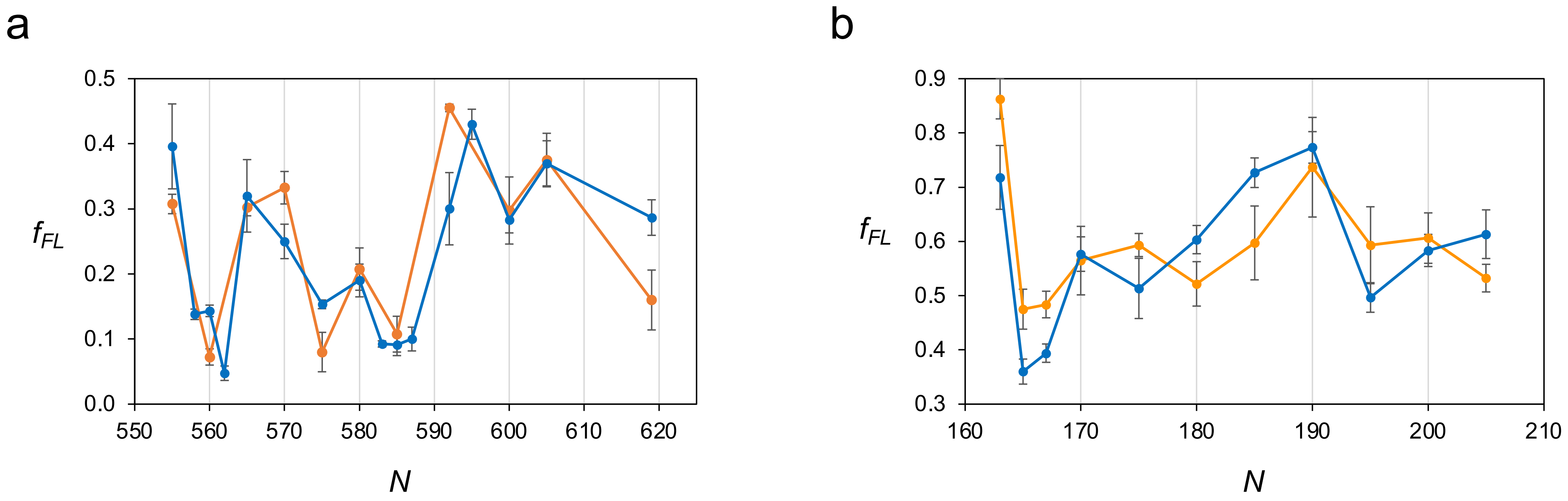

Supplementary Figure 3. (a) Shape overlap between the FLuc FP (blue) and the CTD FP (orange) in the region *N* = 555-619 residues. The f_{FL} values for the latter have been reduced by 0.2 compared to Figure 3. Error bars indicate the standard error of the mean (SEM). (b) Shape overlap between the FLuc FP (blue) and the FLuc+TF FP (orange) in the region *N* = 163-205 residues. The f_{FL} values for the latter have been increased by 0.27 compared to Figure 7. Error bars indicate the standard error of the mean (SEM). Source data are provided in the Source Data file. All data points represent averages for $n \geq 3$ biological replicates, with the exact *n* values given in the Source Data file.

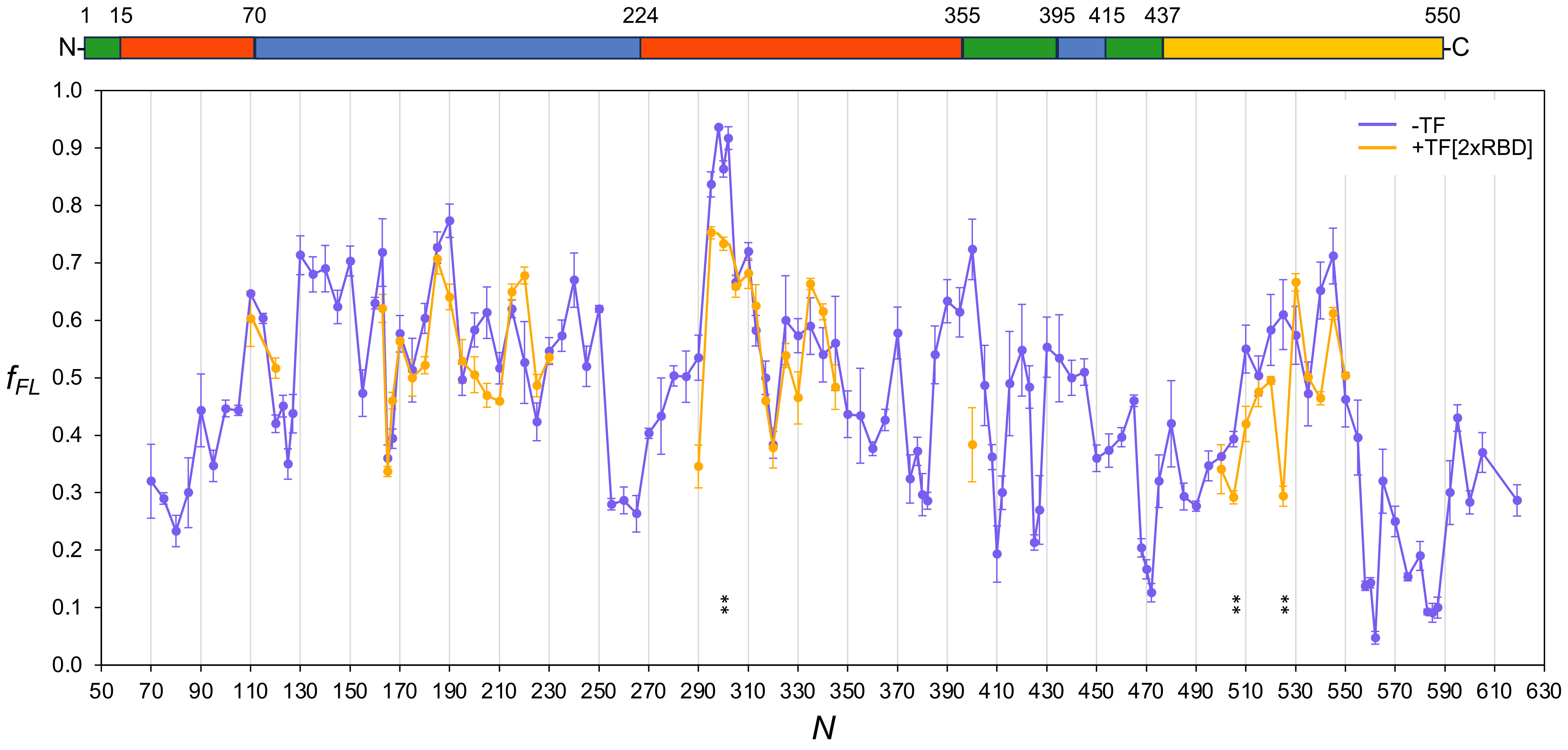

Supplementary Figure 4. Effects of TF[2xRBD] on the FLuc force profile. FLuc FP obtained in the presence of 4 mM TF[2xRBD] (orange), superimposed on the original FP from Figure 3 (blue). All data points represent averages for $n \geq 3$ biological replicates, with the exact n values given in the Source Data file. Constructs for which the difference between the ±TF[2xRBD] f_{FL} values are significant on the $p < 0.01$ (**) level (calculated by a two-sided Student's t-test) are indicated. Error bars indicate the standard error of the mean (SEM).

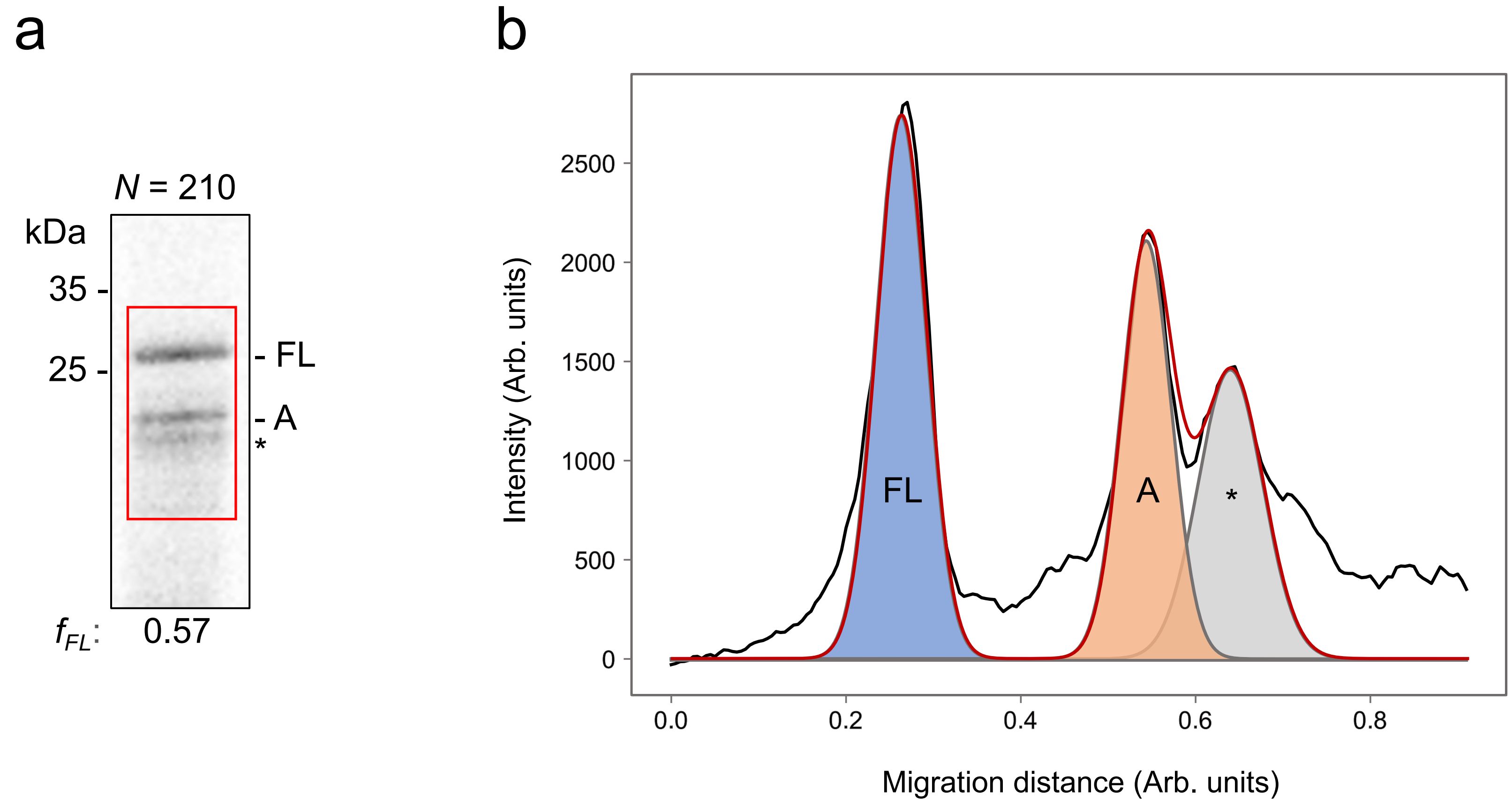

Supplementary Figure 5. Quantitation of SDS-PAGE gels. (a) SDS-PAGE lane for the *N* = 210 residues construct with the quantitated area marked in red. (b) Top-to-bottom intensity profile (in black) of the quantitated area, with Gaussians fit to the three main bands. f_{FL} is calculated as $f_{FL} = I_{FL}/(I_{FL}+I_A)$, where I_A and I_{FL} are the areas under the *A* (in orange) and *FL* (in blue) Gaussians, respectively. The rightmost Gaussian (in grey, band indicated by * in panel a) is most likely generated by ribosomes stacked behind the ribosome arrested on the AP, and is left out of the calculation of f_{FL}. The sum of the Gaussians is indicated by the red curve. The full gel for panel *a* and f_{FL} values for *n* = 3 repeats of the *N* = 210 construct are provided in the Source Data file.

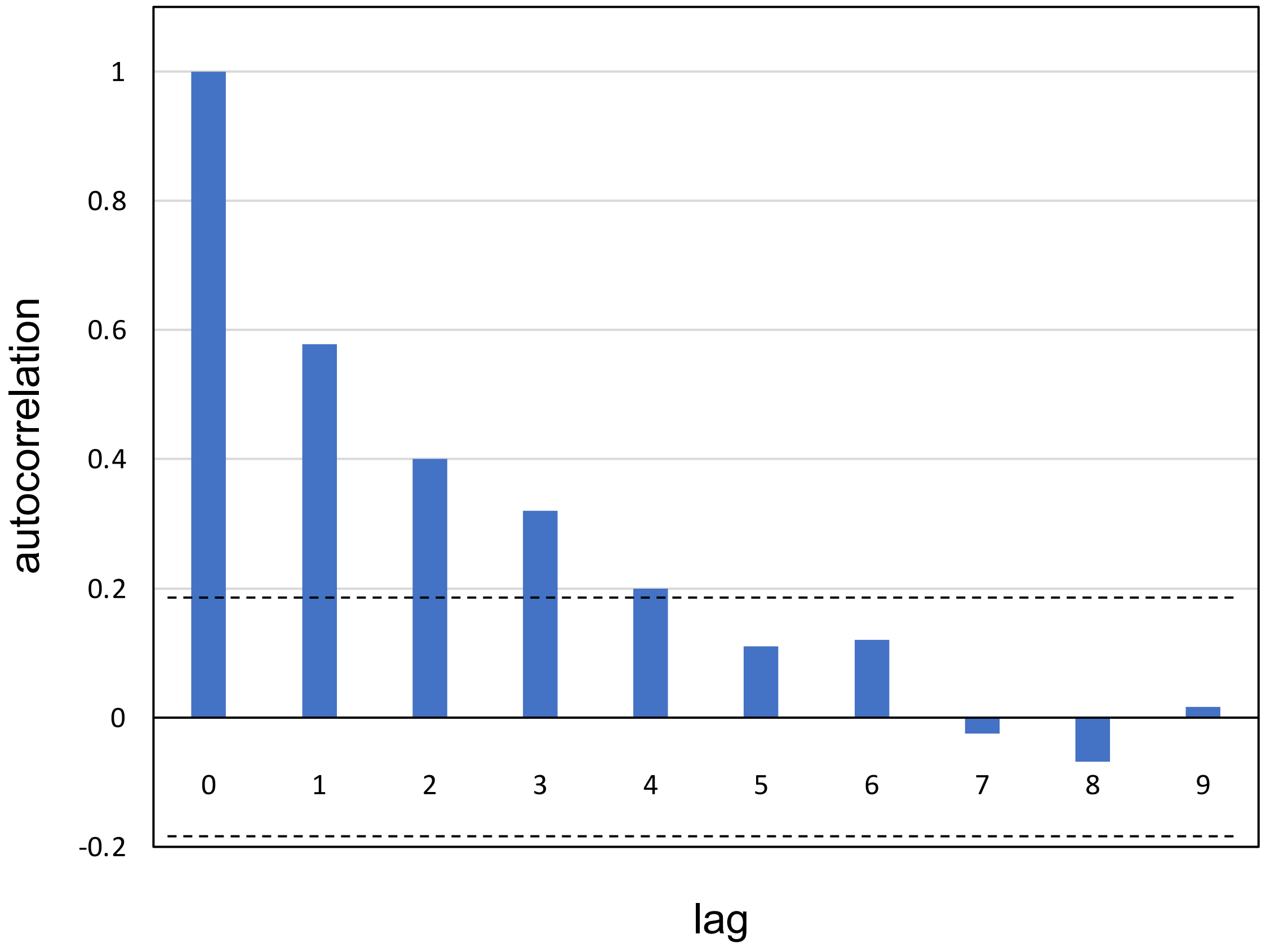

Supplementary Figure 6. Autocorrelation Function calculated for the FLuc FP in Figure 3 (see Methods). The dotted lines indicate the confidence interval ($\pm 2/\sqrt{n}$, where n = 109).

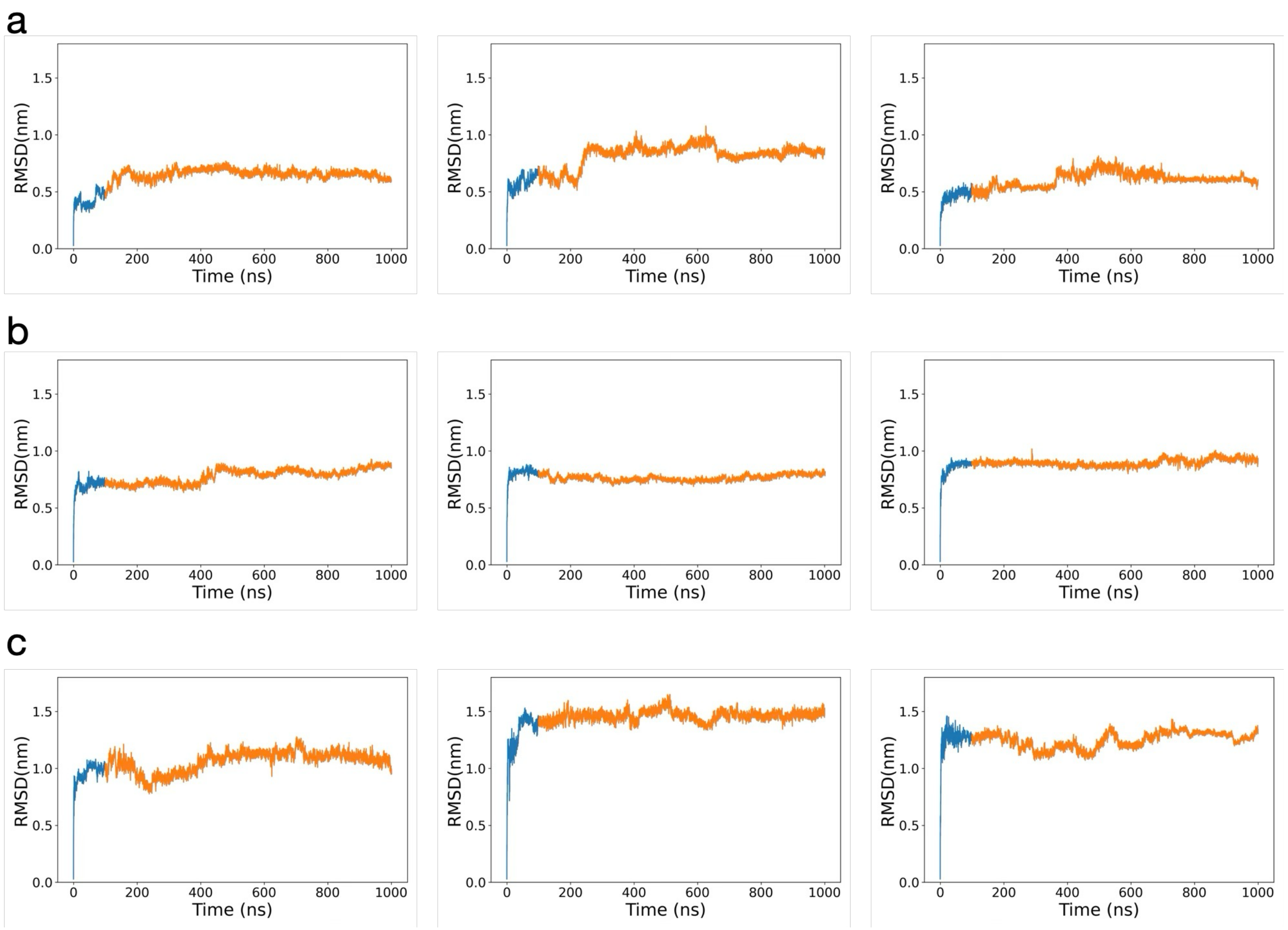

Supplementary Figure 7. RMSD time courses of the MD simulations. RMSD of the C_α atoms to the initial conformation after fitting to the initial conformation is plotted for the three replicas of each system: a) Δ4-80; b) Δ4-110; c) Δ4-150. In all cases, the orange region indicates the section of the trajectory used for analysis.

a

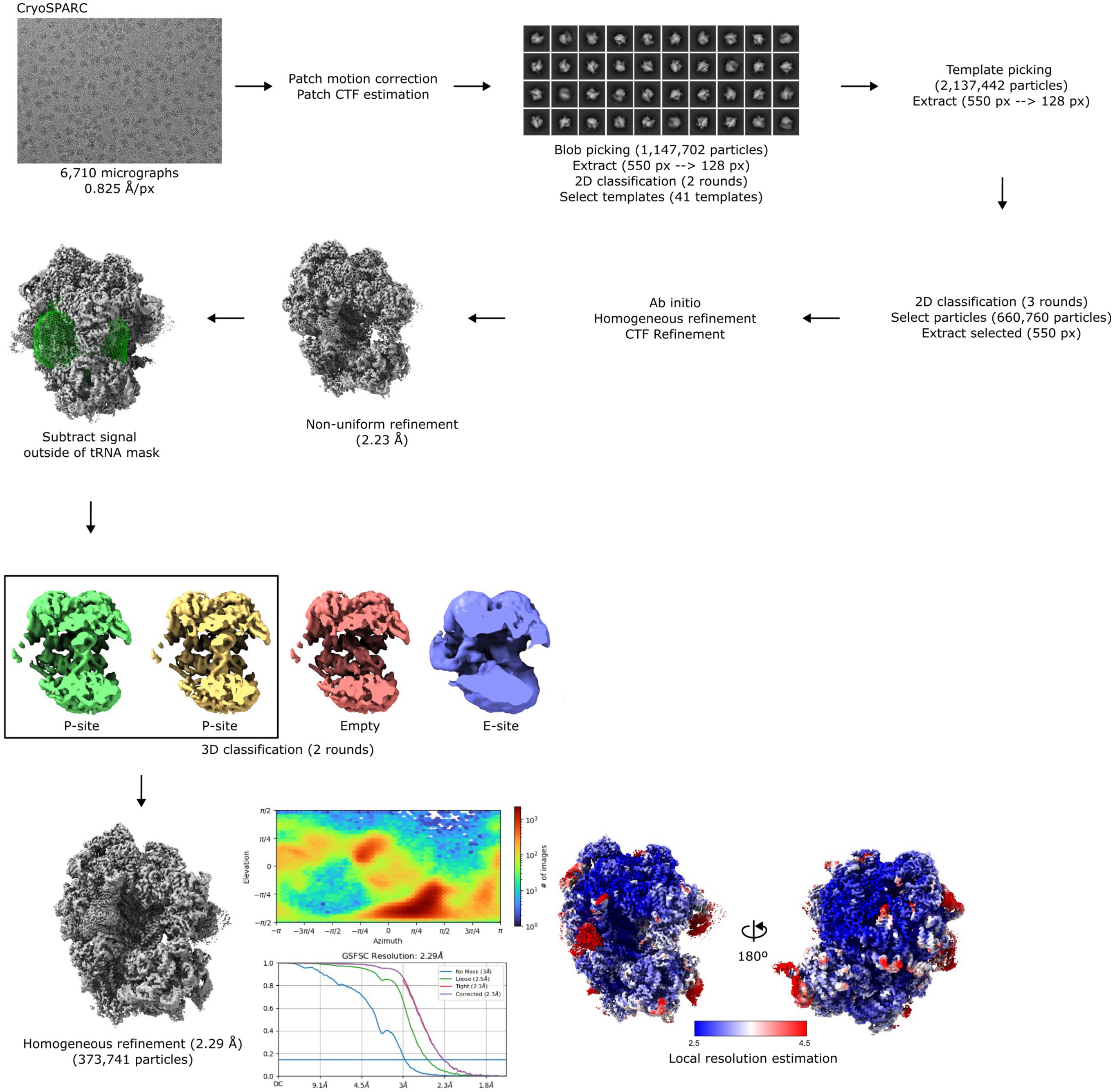
CryoSPARC
6,710 micrographs
0.825 Å/px
Patch motion correction
Patch CTF estimation
Blob picking (1,147,702 particles)
Extract (550 px --> 128 px)
2D classification (2 rounds)
Select templates (41 templates)
Template picking
(2,137,442 particles)
Extract (550 px --> 128 px)
2D classification (3 rounds)
Select particles (660,760 particles)
Extract selected (550 px)
Ab initio
Homogeneous refinement
CTF Refinement
Non-uniform refinement
(2.23 Å)
Subtract signal
outside of tRNA mask
P-site
P-site
Empty
E-site
3D classification (2 rounds)
Homogeneous refinement (2.29 Å)
(373,741 particles)
Elevation
Azimuth
# of images
GSFSC Resolution: 2.29Å
No Mask (3Å)
Loose (2.5Å)
Tight (2.3Å)
Corrected (2.3Å)
180º
2.5
4.5
Local resolution estimation

continued

b

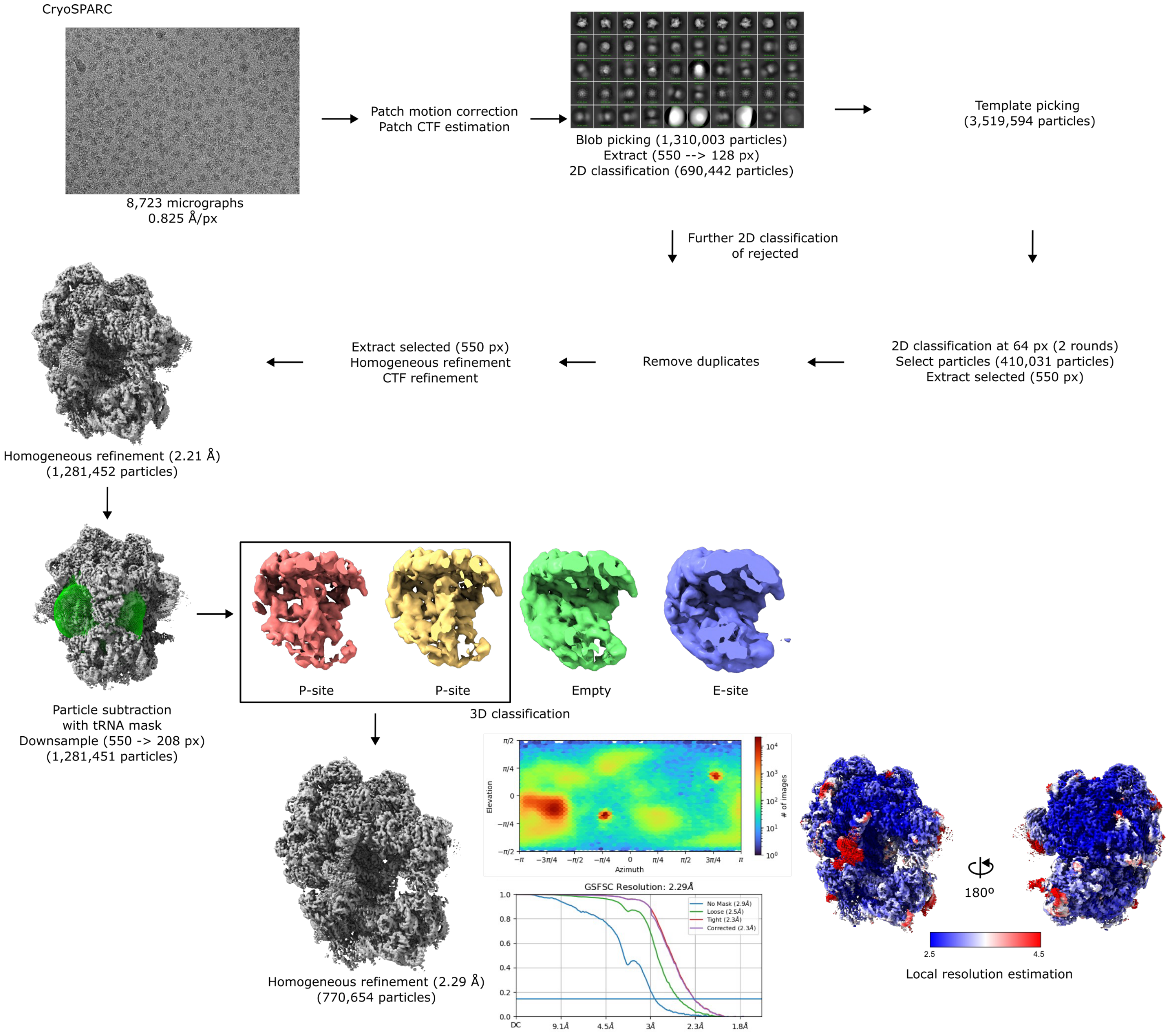
CryoSPARC
8,723 micrographs
0.825 Å/px
Patch motion correction
Patch CTF estimation
Blob picking (1,310,003 particles)
Extract (550 --> 128 px)
2D classification (690,442 particles)
Template picking
(3,519,594 particles)
Further 2D classification
of rejected
2D classification at 64 px (2 rounds)
Select particles (410,031 particles)
Extract selected (550 px)
Remove duplicates
Extract selected (550 px)
Homogeneous refinement
CTF refinement
Homogeneous refinement (2.21 Å)
(1,281,452 particles)
Particle subtraction
with tRNA mask
Downsample (550 -> 208 px)
(1,281,451 particles)
P-site
P-site
Empty
E-site
3D classification
Homogeneous refinement (2.29 Å)
(770,654 particles)
Elevation
Azimuth
# of images
GSFSC Resolution: 2.29Å
No Mask (2.9Å)
Loose (2.5Å)
Tight (2.3Å)
Corrected (2.3Å)
180º
2.5
4.5
Local resolution estimation

continued

c

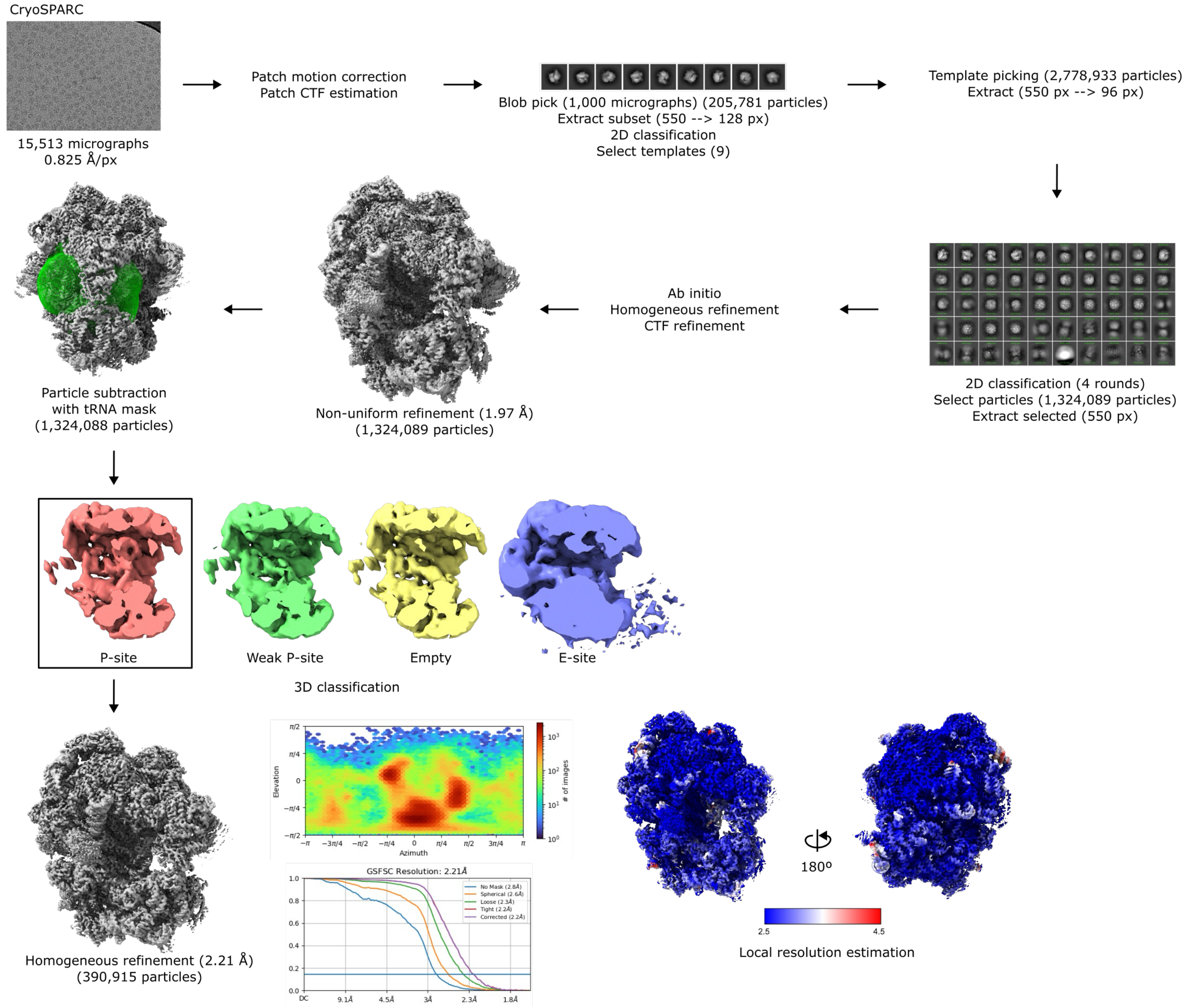

Supplementary Figure 8. Cryo-EM processing pipelines. (a) *N* = 110 residues RNCs. (b) *N* = 130 residues RNCs. (c) *N* = 190 residues RNCs.

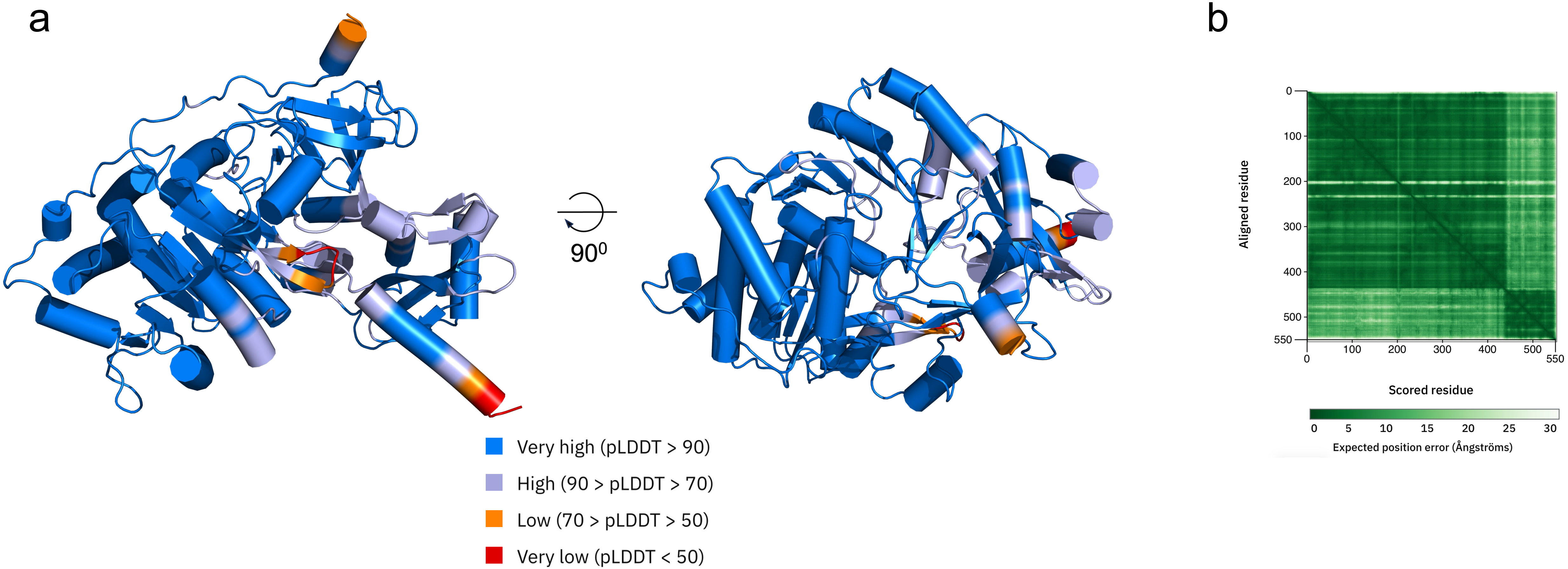

Supplementary Figure 9. (a) AF-P08659-F1 colored according to pLDDT values. (b) Predicted Aligned Errors.

Supplementary Table 1. MD Simulation Setup System Details

	Δ 4-80	Δ 4-110	Δ 4-150
Box dimensions (nm)	a = (8.64151, 0, 0) b= (0,8.64151,0) c= (4.32076,4.32076, 6.11047)	a = (8.64151, 0, 0) b= (0,8.64151,0) c= (4.32076,4.32076, 6.11047)	a = (8.64151, 0, 0) b= (0,8.64151,0) c= (4.32076,4.32076, 6.11047)
Box type	Rhombic dodecahedron	Rhombic dodecahedron	Rhombic dodecahedron
Salt Ions	1 Na^+	1 Cl^-	4 Na^+
Ion concentration	3.64 mM Na^+	3.64 mM Cl^-	14.56 mM Na^+
Total Atoms	44744	44704	44658
Total Waters	13822	13956	14169

Supplementary Table 2. Cryo-EM data collection, refinement and validation statistics

	Escherichia coli ribosome arrested on a chimeric, 110-residue construct featuring a firefly-luciferase truncation followed by SecM 3W (EMDB-56368)	Escherichia coli ribosome arrested on a chimeric, 130-residue construct featuring a firefly-luciferase truncation followed by SecM 3W (EMDB-56453)	Escherichia coli ribosome arrested on a chimeric, 190-residue construct featuring a firefly-luciferase truncation followed by SecM 3W (EMDB-56462)
**Data collection and processing**			
Magnification	105,000x	105,000x	105,000x
Voltage (kV)	300	300	300
Electron exposure ($e^{-}/Å^2$)	40	40	40
Defocus range (μm)	-2.4, -0.6	-2.4, -0.6	-2.4, -0.6
Pixel size (Å)	0.825	0.825	0.825
Symmetry imposed	C1	C1	C1
Initial particle images (no.)	1,147,702	1,310,003	2,778,933
Final particle images (no.)	373,741	770,654	390,915
Map resolution (Å)	2.29	2.29	2.14
FSC threshold	0.143	0.143	0.143
Map resolution range (Å)	1.973 – 39.486	1.800 – 39.566	1.821 – 33.721
