# Supplementary material for: Quasi-continuous cotranslational compaction and folding of a multidomain protein
Source: Nat Commun. 2026 Oct 5;17:10495. doi: 10.1038/s41467-026-78090-y (PMC13639035; doi:10.1038/s41467-026-78090-y)
Supplement: Supplementary file 2 [file 41467_2026_78090_MOESM2_ESM.pdf]

Supplementary Data 1

Amino acid sequences of all constructs used in this work.

Supplementary Movie 1

MD simulations carried out for FLuc fragments D4-80. The movie contains three independent production trajectories, each 1 µs, concatenated end to end. The trajectory name (Δ4-80 Trajectory 1-3) appears in the lower-left corner, and the simulation time for each trajectory appears in the lower-right corner. The protein is shown in cartoon representation and is colored as follows: N-terminal region (residues 81-191) in cyan, β-hairpin (residues 192-211) in gray, and C-terminal region (residues 212-282) in red. Solvent and ions are omitted for clarity.

Supplementary Movie 2

MD simulations carried out for FLuc fragments D4-110. The movie contains three independent production trajectories, each 1 µs, concatenated end to end. The trajectory name (Δ4-110 Trajectory 1-3) appears in the lower-left corner, and the simulation time for each trajectory appears in the lower-right corner. The protein is shown in cartoon representation and is colored as follows: N-terminal region (residues 111-191) in cyan, β-hairpin (residues 192-211) in gray, and C-terminal region (residues 212-282) in red. Solvent and ions are omitted for clarity.

Supplementary Movie 3

MD simulations carried out for FLuc fragments D4-150. The movie contains three independent production trajectories, each 1 µs, concatenated end to end. The trajectory name (Δ4-150 Trajectory 1-3) appears in the lower-left corner, and the simulation time for each trajectory appears in the lower-right corner. The protein is shown in cartoon representation and is colored as follows: N-terminal region (residues 151-191) in cyan, β-hairpin (residues 192-211) in gray, and C-terminal region (residues 212-282) in red. Solvent and ions are omitted for clarity.
