# Supplementary material for: Quasi-continuous cotranslational compaction and folding of a multidomain protein
Source: Nat Commun. 2026 Oct 5;17:10495. doi: 10.1038/s41467-026-78090-y (PMC13639035; doi:10.1038/s41467-026-78090-y)
Supplement: Supplementary file 5 [file 41467_2026_78090_MOESM8_ESM.pdf]

nature portfolio

Peer Review File

Quasi-continuous cotranslational compaction and folding of a multidomain protein

Corresponding Author: Dr Gunnar von Heijne

This file contains all reviewer reports in order by version, followed by all author rebuttals in order by version.

Version 0:

Reviewer comments:

Reviewer #1

(Remarks to the Author)
In their manuscript "Quasi-continuous cotranslational compaction and folding
of a multidomain protein", Mitsikosta et al. applied an extensive force profile analysis to the cotranslational folding of the multidomain protein FLuc. They found high-force events likely representing domain folding and low-force events representing stable intermediates. In elegant experiments, they were able to narrow down the region crucial for the folding event corresponding to the highest peak by either deleting N-terminal residues or replacing C-terminal residues by GS-linkers, highlighting that the method can be used to obtain a high level of detail of cotranslational folding.

Throughout the manuscript, large f_FL values are attributed to compaction or folding. However, interactions of the nascent chain with the exit tunnel (e.g. via electrostatics) could also lead to pulling force on the peptide which could result in release of stalling. How do the authors rule out such effects? If that cannot be ruled out, this should be discussed.

The authors applied the Jacobs and Shakhnovic method to predict intermediates. In the methods I could not find any details on how the method was applied. Is the method so straightforward that no details need to be reported?

Regarding Fig. 3: I think it would be beneficial to link the subdomain boundaries to structure and/or sequence (as presented in Fig. 1). As it is presented right now, it is hard to identify what parts are identified as subdomains.

Regarding Fig. 4: if residues directly upstream of the (17aa long) AP are replaced, I do not understand why the replacements start at residue 280 and not 283.

Fig. 5b should have a color bar for the f_FL values.

On page 9, the authors write "For all three constructs, stalled NCs were readily apparent in the ET, with only small compact densities evident near the uL24 loop in the exit port, Fig. 6. The absence of more extensive folded domains in the ET shows that the FP peaks are not generated by the formation of local substructures within the ET."
The absence of compact densities does not rule out folded domains, since if folded domains were mobile in the tunnel their density would be smeared out and therefore not visible.

Regarding Fig S1: It is not clear what is plotted here, the y axis does not have a label (just units). Why is there only one curve, if two structures were used. Structures should be explained (i.e. crystal structure of the Luciferase) rather than just providing PDB ids. I did not find references for the two structures, they should be included!

For the MD simulations, why were only counter ions added to the system instead of adding salt at concentrations corresponding to the experiments?

typos:
p.9 "strcutures"
p. 12 "This in agreement…" -> "This is in agreement…"

Reviewer #2

(Remarks to the Author)
Overall I thought this was a very interesting paper, which would be a good fit for Nature Communications. In particular the quasi-continuous folding during translation is notable, fitting with recent other findings, and a specific strength of the technique used. The question of co-translational folding is of increasing and broad interest, linking diverse cellular processes. I did however have a number of concerns that would be important to address for the paper to be publishable, in my opinion.
Critical points in no specific order:
1) In the main text and figure descriptions it is never explicitly stated, that in all 5-residue deletion constructs, a minimum of 3 amino acid GS linker remains between the AP and the FLuc sequence. This is slightly confusing and could be easily resolved by adding a not to the text or potentially adapting a figure to enhance clarity. It might also be interesting to know why it was chosen to leave a minimum of 3 amino acids in that position.
2) This is a central sentence: 'This further implies that the intermediate-amplitude region in the -TF FP from N = 130-250 residues reflects a continuous compaction/folding process, rather than an abrupt, cooperative folding event.', as it introduces the notion of quasi-continuous folding that is also in the title. However this sentence merely says that this notion is 'implied'. If the authors think this is a main result that indeed should be in the title and abstract as a main finding, then is would be important to discuss this conclusion in more detail. Could there be alternative explanations of the data, and why would they be unlikely? Why is it notable that the fFL goes up and then remains up? Has this been observed in previous fFL measurements on other proteins, or does one then see the more expected peaks that do not remain high? I am not looking for a 100% proof, as this can be rarely the case, but the reader now remains a bit in the dark about what the data actually say in terms of folding mechanism.
3) It would be good to discuss the limitations of the method more clearly, including the lack of active translation, the possible differences between the measured quantity and folding, and the lack of folding dynamics insights.
4) While the authors do a reasonable job in discussing previous work, this can still be improved. Please discuss where results match or not with recent co-translational luciferase study by the Balchin lab, while also recent studies showing that TF co-translationally binds partial folds of DHFR (Till, PNAS 2025) and across the proteome (Galmozzi, Nat. Comm. 2025) is relevant to the discussion in the TF section and the discussion, and also are arguably the closest to supporting the idea of quasi-continuous folding or compaction, even as the present work shows this more directly.
5) In addition, do the presented results not argue against binding partial folds, as TF decreases the force? If that is the case, what could be the cause of these differences with previous literature? This should be clearly discussed and argued through.
6) Page 6 indicates that SWORD2 boundaries 'correlate quite well', but this was not so easy to judge when looking at the data. Such statements should be based on a statistical analysis. Other expressions also could be more precise. For instance just below it indicates a peak at 130 residues, but this number seems very approximate.
7) To me the role of the cryo-EM is unclear. The fact that no structures are observed in the ET is not very notable and seems to be the only conclusion. I presume the authors aimed to see more nascent structure – then it would be better to just openly discuss that and why this was not realized. Alternatively one could remove this as a separate section and limit it to a single sentence in another section.
8) TF[2xRBD] data is not discussed in a manner that significantly adds to the point of the paper. Could these experiments be further analyzed or left out?
9) Page 9/Paragraph 2/Line 4: The typo "strcutures" is likely meant to say „structures".
10) It is quite interesting to see that the SDS PAGE shows a significant amount of arrested peptides that also have a following ribosome there. Is there any significance to the other bands below? Are they due to truncated peptides or other factors?
11) Page 3/Paragraph 3/Line 5: When referring to "a handful of studies" citing these specific studies instead of a review paper might be nice.
12) Page 17/Paragraph Cryo-EM: "according to 77" could be elevated by specifically mentioning the paper in the text.
13) I found the figures very rudimentary, including but not limited to unequal font sizes, and a very minimal figure 8 that I would not say warrants a separate figure, and would benefit from more visual explanation.
- Fig. 5: The lower bound of the confidence interval is not visible, which would be nice. Additionally, the 0-9 labels on the x-axis could be moved lower (potentially below the x-axis) so that the data is not obstructed.
- Fig.7: In some instances the FP shows higher fFL for the TF condition(N~120, 220, 340, 460500), suggesting a potentially different TF binding mechanism to what is proposed for stretches with lowered fFL. This could be discussed more in-depth.
- Fig. 5: Labelling the β1-4 would help with understanding which part of the structure is being referred to.
- Fig. 5: Adding a legend with a color bar would make interpreting the figure easier.
- Suppl. Fig. S2: In the description "top panel" should be explained as referring to S2c, as it is also done right below for "bottom panel".

Reviewer #3

(Remarks to the Author)
In the manuscript by Mitsikosta and colleagues, the authors use a Force Profile Analysis (FPA) approach, combined with cryo-EM and MD simulations, to probe the folding landscape of a multidomain protein as it is synthesized on the ribosome. The major question the authors address is how cotranslational folding of a multidomain protein on the ribosome occurs, and what role chaperones play in the process. Here they use a bacterial translation system that allows them to add or leave out Trigger Factor (TF) as the chaperone. The authors observe a complex pattern of intermediate forces with one major peak (high force) and multiple troughs (low force) interspersed. Trigger Factor generally engages the nascent protein chain along most its length apart from the very C-terminus. The authors do not observe an ordered folding intermediate in the ribosome exit tunnel vestibule region in cryo-EM maps. However, MD simulations suggest a sizeable fragment of the N-terminal

domain of Luciferase can form somewhat stable folded forms, in the absence of the complete sequence of the isolated RF-2 domain. The authors interpret these data to indicate the nascent chain undergoes a series of compaction events along the folding trajectory, with one major folding transition at the peak force. This pattern is distinct from single-domain proteins, which tend to show what might be considered more classical 2-state behavior.

Overall, the experiments are technically sound, but there are some aspects of the interpretation and discussion that could be expanded to make the article of more general interest. For example, the authors do not really address alternative interpretations of their data. How are we to understand the compaction the authors propose? Are these quasi-stable intermediates, or could the nascent chain be sampling folded and unfolded states on a rapid timescale, a more kinetic interpretation? In vitro translation is rather slow compared to that in cells. Is this something that may impact the interpretation of the results? Additionally, how do their data compare to studies of single-domain proteins that show the ribosome destabilizes folding in close proximity to the ribosome surface? Also, the authors note that others have probed the folding of multidomain proteins on the ribosome (i.e. reviewed in ref. 13), but don’t really spend any time in the Discussion comparing their results with Luciferase with the other studies. Are there general concepts that emerge?

The authors could also improve the manuscript with the following changes and analyses.

1. The cryo-EM maps should be shown with a low-pass filter, to see if there is any more extensive density outside the ribosome exit tunnel. It looks like the box size the authors used for particle picking (550 px at 0.825 Å per px) should be sufficient to cover that region. (It would help to expand the materials and methods on the cryoEM processing, to be clear on this point.) Perhaps there is more to be seen that would inform the view of compaction the authors propose.

2. In Figure 2b, indicate how the “A” band is boxed, given the smear below.

3. Figure 3 is hard to follow, without a key for the labels on the actual figure.

4. Figure 5 needs a color scale bar on the actual figure to understand the coloring scheme.

5. Figure 7 also needs a key on the figure, as well as Supplemental Figure S4.

6. Each panel in Supplemental Figure S6 has a lot of white space around each graphic. It would help to blow these up as much as possible to fill the rectangular space.

Version 1:

Reviewer comments:

Reviewer #1

(Remarks to the Author)
The authors have addressed all of my concerns sufficiently.

Reviewer #2

(Remarks to the Author)
I am now satisfied with the reply and revisions, and support publication.

Reviewer #3

(Remarks to the Author)
The authors have addressed the reviewers' questions and suggestions. In particular I appreciate the addition of Supplemental Figure S5 and the git repository information in the Methods for quantification of bands in the SDS gels.

Open Access This Peer Review File is licensed under a Creative Commons Attribution 4.0 International License, which permits use, sharing, adaptation, distribution and reproduction in any medium or format, as long as you give appropriate credit to the original author(s) and the source, provide a link to the Creative Commons license, and indicate if changes were made.
In cases where reviewers are anonymous, credit should be given to 'Anonymous Referee' and the source.
The images or other third party material in this Peer Review File are included in the article's Creative Commons license, unless indicated otherwise in a credit line to the material. If material is not included in the article's Creative Commons license and your intended use is not permitted by statutory regulation or exceeds the permitted use, you will need to obtain permission directly from the copyright holder.
To view a copy of this license, visit https://creativecommons.org/licenses/by/4.0/

POINT-BY-POINT RESPONSE TO REVIEWER COMMENTS

Reviewer #1 (Remarks to the Author):

In their manuscript "Quasi-continuous cotranslational compaction and folding of a multidomain protein", Mitsikosta et al. applied an extensive force profile analysis to the cotranslational folding of the multidomain protein FLuc. They found high-force events likely representing domain folding and low-force events representing stable intermediates. In elegant experiments, they were able to narrow down the region crucial for the folding event corresponding to the highest peak by either deleting N-terminal residues or replacing C-terminal residues by GS-linkers, highlighting that the method can be used to obtain a high level of detail of cotranslational folding.

Throughout the manuscript, large fFL values are attributed to compaction or folding. However, interactions of the nascent chain with the exit tunnel (e.g. via electrostatics) could also lead to pulling force on the peptide which could result in release of stalling. How do the authors rule out such effects? If that cannot be ruled out, this should be discussed.

- The 20 C-terminal AP residues (including an N-terminal SGS tripeptide) are constant in all constructs, hence differential ribosome interactions between constructs would be restricted to ~15-20 residues in the exit tunnel vestibule, which according to current knowledge will at best have a marginal influence on f_{FL}. We have added a discussion of this point to the Ms.

The authors applied the Jacobs and Shakhnovic method to predict intermediates. In the methods I could not find any details on how the method was applied. Is the method so straightforward that no details need to be reported?

- We've added a new section to Methods where we briefly explain the method. The theory and implementation details are described in the cited References.

Regarding Fig. 3: I think it would be beneficial to link the subdomain boundaries to structure and/or sequence (as presented in Fig. 1). As it is presented right now, it is hard to identify what parts are identified as subdomains.

- Thanks you for the suggestion! The subdomain boundaries are now plotted above the FP graph in Fig. 3, Fig. 7, and Fig. S4 in such a way that the subdomain boundaries match the *N*-values where the boundary has just emerged from the ET. This addition makes the SWORD2 domain boundaries superfluous, and they have consequently been removed from the Ms.

Regarding Fig. 4: if residues directly upstream of the (17aa long) AP are replaced, I do not understand why the replacements start at residue 280 and not 283.

- As now better explained in the Ms, for simplicity we count the SGS tripeptide

present in all constructs as part of the AP which therefore is 20 residues long.

Fig. 5b should have a color bar for the fFL values.

- Color bar has been added.

On page 9, the authors write “For all three constructs, stalled NCs were readily apparent in the ET, with only small compact densities evident near the uL24 loop in the exit port, Fig. 6. The absence of more extensive folded domains in the ET shows that the FP peaks are not generated by the formation of local substructures within the ET.”
The absence of compact densities does not rule out folded domains, since if folded domains were mobile in the tunnel their density would be smeared out and therefore not visible.

- In principle the reviewer is correct. We’ve added a disclaimer saying that we can’t completely rule out that a folded substructure is bouncing around in the vestibule, though we consider this unlikely.

Regarding Fig S1: It is not clear what is plotted here, the y axis does not have a label (just units). Why is there only one curve, if two structures were used. Structures should be explained (i.e. crystal structure of the Luciferase) rather than just providing PDB ids. I did not find references for the two structures, they should be included!

- We have expanded the Y-axis label. Two structures are used in the calculation, producing one curve as now explained in the new Methods section. Structures are now better described (also in Methods) and structure citations have been added.

For the MD simulations, why were only counter ions added to the system instead of adding salt at concentrations corresponding to the experiments?

- The *in vitro* translation experiments were performed in the PURExpress system buffer (9 mM magnesium acetate, 5 mM potassium phosphate, 95 mM potassium glutamate, 5 mM NH_4Cl, 1 mM spermidine, 8 mM putrescine, 1 mM DTT), which is required for proper ribosome function. However, in our MD simulations, we sought to assess the conformational states that the three FLuc constructs could sample on a microsecond timescale, in the absence of the ribosome. Thus, we opted for a simplified setup and neutralized the system with the minimum number of Na^+ or Cl^- ions required (new Supplemental Table S2). For the system Δ4-80, this corresponded to a concentration of 3.64 mM Na^+; for the system Δ4-110, it was 3.64 mM Cl^-; and for the system Δ4-150, it was 14.56 mM Na^+. We have added the following sentences to the Methods Section: “The systems were neutralized with the minimum number of Na^+ or Cl^- ions required. As we simulated the constructs in the absence of a ribosome, we did not use the same

experimental conditions as in the Force Profile Analysis. Supplemental Table S2 summarizes the composition of each system, including box dimensions and total atom counts."

typos:
p.9 "strcutures"
p. 12 "This in agreement…" -> "This is in agreement…"

- Corrected

Reviewer #2 (Remarks to the Author):

Overall I thought this was a very interesting paper, which would be a good fit for Nature Communications. In particular the quasi-continuous folding during translation is notable, fitting with recent other findings, and a specific strength of the technique used. The question of co-translational folding is of increasing and broad interest, linking diverse cellular processes. I did however have a number of concerns that would be important to address for the paper to be publishable, in my opinion.
Critical points in no specific order:

1) In the main text and figure descriptions it is never explicitly stated, that in all 5-residue deletion constructs, a minimum of 3 amino acid SGS linker remains between the AP and the FLuc sequence. This is slightly confusing and could be easily resolved by adding a not to the text or potentially adapting a figure to enhance clarity. It might also be interesting to know why it was chosen to leave a minimum of 3 amino acids in that position.

- As now better explained in the Ms, for simplicity we count the SGS tripeptide – which is included as an "insulator" between the AP and FLuc – as part of the AP which therefore is counted as being 20 residues long.

2) This is a central sentence: 'This further implies that the intermediate-amplitude region in the -TF FP from N = 130-250 residues reflects a continuous compaction/folding process, rather than an abrupt, cooperative folding event.', as it introduces the notion of quasi-continuous folding that is also in the title. However this sentence merely says that this notion is 'implied'. If the authors think this is a main result that indeed should be in the title and abstract as a main finding, then is would be important to discuss this conclusion in more detail. Could there be alternative explanations of the data, and why would they be unlikely? Why is it notable that the fFL goes up and then remains up? Has this been observed in previous fFL measurements on other proteins, or does one then see the more expected peaks that do not remain high? I am not looking for a 100% proof, as this can be rarely the case, but the reader now remains a bit in the dark about what the data actually say in terms of folding mechanism.

- We have tried to clarify the arguments in favor of our hypothesis that medium-

amplitude parts of the FP represent compaction, not only in the Discussion but also when we describe the FLuc FP, the cryo-EM data, and the TF results. Although we do not conclusively prove ‘quasi-continuous compaction’, we feel that, collectively, the data provide quite strong support for this idea.

3) It would be good to discuss the limitations of the method more clearly, including the lack of active translation, the possible differences between the measured quantity and folding, and the lack of folding dynamics insights.

- This is a good point. We have added a paragraph on p. 5.

4) While the authors do a reasonable job in discussing previous work, this can still be improved. Please discuss where results match or not with recent co-translational luciferase study by the Balchin lab, while also recent studies showing that TF co-translationally binds partial folds of DHFR (Till, PNAS 2025) and across the proteome (Galmozzi, Nat. Comm. 2025) is relevant to the discussion in the TF section and the discussion, and also are arguably the closest to supporting the idea of quasi-continuous folding or compaction, even as the present work shows this more directly.

- Thank you for alerting us to these papers, which are obviously of relevance. Citations have been added.

5) In addition, do the presented results not argue against binding partial folds, as TF decreases the force? If that is the case, what could be the cause of these differences with previous literature? This should be clearly discussed and argued through.

- Previous work has shown that TF reduces the amplitude of the early part of the final folding peak for DHFR (Nilsson, 2016), and also tends to reduce fFL in a couple of other proteins (Chen, 2025). This can be explained by TF stabilizing compact but not fully folded states, thus competing with the final folding step (and release from TF). In the Ms, we argue that, because the TF binding crevice is distant from the exit port, there is competition between compaction/folding at the exit site and compaction+TF biding some 20 residues away from the exit port. Thus, TF might reduce compaction at the exit site while favoring it some distance away, from which pulling forces may be less efficiently transmitted to the AP.

6) Page 6 indicates that SWORD2 boundaries ‘correlate quite well’, but this was not so easy to judge when looking at the data. Such statements should be based on a statistical analysis. Other expressions also could be more precise. For instance just below it indicates a peak at 130 residues, but this number seems very approximate.

- As suggested by the Referee #1, we have now included the subdomain graphic from Fig. 1 in both Fig 3 and Fig 7, so the SWORD2 boundaries are no longer needed and have been removed (together with the ‘correlate quite well’ statement).

7) To me the role of the cryo-EM is unclear. The fact that no structures are observed in the ET is not very notable and seems to be the only conclusion. I presume the authors aimed to see more nascent structure – then it would be better to just openly discuss that and why this was not realized. Alternatively one could remove this as a separate section and limit it to a single sentence in another section.

- The referee is right about why these experiments were done, and we have now stated this in the Ms. The fact that we see no folded structures in the ET is important in that it supports our hypothesis that the medium-amplitude portions of the FP reflect compaction outside the ribosome. Even though it's only a short section, we think that it makes more sense to keep it separate rather than including these results in some other section.

8) TF[2xRBD] data is not discussed in a manner that significantly adds to the point of the paper. Could these experiments be further analyzed or left out?

- We think that the observation of the strong effects on the FP seen with both TF and TF[2xRBD] at N = 300 and N = 400 residues, despite the much longer distance to the canonical TF biding cavity in TF[2xRBD], are worth including because they may imply an interaction between the NC and the RBD rather than the binding cavity. Obviously, this is not a major point, but we feel that it adds enough to the Ms to warrant inclusion as a Supplemental Figure.

9) Page 9/Paragraph 2/Line 4: The typo "strcutures" is likely meant to say „structures".

- Corrected

10) It is quite interesting to see that the SDS PAGE shows a significant amount of arrested peptides that also have a following ribosome there. Is there any significance to the other bands below? Are they due to truncated peptides or other factors?

- One or more bands immediately below the *A* product are always seen in these types of experiments and most likely indicate ribosomes stacked behind the arrested ribosome. They are not included in the quantitation of the FL and A bands (see Methods and Supplemental Fig. S5), and hence do not affect the f_{FL} values. Faint bands seen at lower Mw in Fig. 2b do not vary in size between the different constructs and hence represent unrelated background bands produced in PURExpress, or weak translational pause sites (c.f. Fig. 1 in Biochemistry 57:3460); this is now noted in the legend to Fig. 2.
-

11) Page 3/Paragraph 3/Line 5: When referring to "a handful of studies" citing these specific studies instead of a review paper might be nice.

- Citations have been included.

12) Page 17/Paragraph Cryo-EM: "according to 77" could be elevated by specifically

mentioning the paper in the text.

- Citation now included in the main text.

13) I found the figures very rudimentary, including but not limited to unequal font sizes, and a very minimal figure 8 that I would not say warrants a separate figure, and would benefit from more visual explanation.

- We have tried to improve the consistency of the Figures, but are grateful for any additional suggestions. We have removed Fig. 8.

- Fig. S5: The lower bound of the confidence interval is not visible, which would be nice. Additionally, the 0-9 labels on the x-axis could be moved lower (potentially below the x-axis) so that the data is not obstructed.

- We have moved the labels to a more legible position and have included the lower bound of the confidence interval.

- Fig.7: In some instances the FP shows higher fFL for the TF condition (N~120, 220, 340, 460500), suggesting a potentially different TF binding mechanism to what is proposed for stretches with lowered fFL. This could be discussed more in-depth.

- A few such instances where TF increases rather than decreases fFL are also evident in Chen et al (2025); this study is now cited in the Ms. Speculating, N=125 could perhaps be the first compaction event at TF (~80 residues emerged from the ET), stabilizing a compacted state that is not stable when not bound to TF. At N=365, the NC is ~60 residues longer than when the 80-260 intermediate forms, so maybe the increase in fFL +TF again represents TF binding to the first compacted state formed from residues beyond the stable N=300 intermediate. This suggestion is, however, based on very limited data and we would rather refrain from such speculations in the Ms.

- Fig. 5: Labelling the β1-4 would help with understanding which part of the structure is being referred to.

- Labels have been added.

- Fig. 5: Adding a legend with a color bar would make interpreting the figure easier.

- Color bar has been added.

- Suppl. Fig. S2: In the description “top panel” should be explained as referring to S2c, as it is also done right below for “bottom panel”.

- Corrected.

Reviewer #3 (Remarks to the Author):

In the manuscript by Mitsikosta and colleagues, the authors use a Force Profile Analysis (FPA) approach, combined with cryo-EM and MD simulations, to probe the folding landscape of a multidomain protein as it is synthesized on the ribosome. The major question the authors address is how cotranslational folding of a multidomain protein on the ribosome occurs, and what role chaperones play in the process. Here they use a bacterial translation system that allows them to add or leave out Trigger Factor (TF) as the chaperone. The authors observe a complex pattern of intermediate forces with one major peak (high force) and multiple troughs (low force) interspersed. Trigger Factor generally engages the nascent protein chain along most its length apart from the very C-terminus. The authors do not observe an ordered folding intermediate in the ribosome exit tunnel vestibule region in cryo-EM maps. However, MD simulations suggest a sizeable fragment of the N-terminal domain of Luciferase can form somewhat stable folded forms, in the absence of the complete sequence of the isolated RF-2 domain. The authors interpret these data to indicate the nascent chain undergoes a series of compaction events along the folding trajectory, with one major folding transition at the peak force. This pattern is distinct from single-domain proteins, which tend to show what might be considered more classical 2-state behavior.

Overall, the experiments are technically sound, but there are some aspects of the interpretation and discussion that could be expanded to make the article of more general interest. For example, the authors do not really address alternative interpretations of their data. How are we to understand the compaction the authors propose? Are these quasi-stable intermediates, or could the nascent chain be sampling folded and unfolded states on a rapid timescale, a more kinetic interpretation? In vitro translation is rather slow compared to that in cells. Is this something that may impact the interpretation of the results? Additionally, how do their data compare to studies of single-domain proteins that show the ribosome destabilizes folding in close proximity to the ribosome surface? Also, the authors note that others have probed the folding of multidomain proteins on the ribosome (i.e. reviewed in ref. 13), but don’t really spend any time in the Discussion comparing their results with Luciferase with the other studies. Are there general concepts that emerge?

- These are good questions, but at present we can only address them in outline.

  Concerning the nature of the “compacted” states, the cited HDX-MS results suggest that they are highly dynamic rather than quasi-stable. We have added a reference to possible electrostatic interactions with the ribosome surface.

  We have added a comment that there are no obvious counterparts to the extensive medium-amplitude sections and the deep dips seen in the FLuc FP in the few multi-domain proteins studied by FPA or other methods to date (Chen 2020, 2025). Given the paucity of examples, it is not really possible to generalize

much beyond the fact that (some) subdomains fold cotranslationally into their native structure, with details depending on the overall architecture/topology of the whole protein, and we would rather refrain from too much speculation

The authors could also improve the manuscript with the following changes and analyses.

1. The cryo-EM maps should be shown with a low-pass filter, to see if there is any more extensive density outside the ribosome exit tunnel. It looks like the box size the authors used for particle picking (550 px at 0.825 Å per px) should be sufficient to cover that region. (It would help to expand the materials and methods on the cryoEM processing, to be clear on this point.) Perhaps there is more to be seen that would inform the view of compaction the authors propose.

- After filtering to 5 or 7Å, we do see weak density from the TF “foot” near the exit portal, but see no extended density (TF or otherwise) outside the ribosome. Because the TF density is so weak, we don’t think that it is worth mentioning in the Ms. We’ve included the box size in the Methods, as suggested.

2. In Figure 2b, indicate how the “A” band is boxed, given the smear below.

- Quantitation is not done by “boxing” the bands, but by fitting Gaussians to the density profile as described in Methods (and now shown in Supplemental Fig. S5).

3. Figure 3 is hard to follow, without a key for the labels on the actual figure.

- Keys now included on the Figure

4. Figure 5 needs a color scale bar on the actual figure to understand the coloring scheme.

- Color bar added

5. Figure 7 also needs a key on the figure, as well as Supplemental Figure S4.

- Keys added to both Figures.

6. Each panel in Supplemental Figure S6 has a lot of white space around each graphic. It would help to blow these up as much as possible to fill the rectangular space.

- We have increased the size and resolution so that the Figure (now called Fig. S8) can be blown up on a screen for detailed viewing.
